# Tyk2 is a tumor suppressor in colorectal cancer

**DOI:** 10.1080/2162402X.2022.2127271

**Published:** 2022-09-26

**Authors:** Stefan Moritsch, Bernadette Mödl, Irene Scharf, Lukas Janker, Daniela Zwolanek, Gerald Timelthaler, Emilio Casanova, Maria Sibilia, Thomas Mohr, Lukas Kenner, Dietmar Herndler-Brandstetter, Christopher Gerner, Mathias Müller, Birgit Strobl, Robert Eferl

**Affiliations:** a Center for Cancer Research, Medical University of Vienna & Comprehensive Cancer Center, Vienna, Austria; b Department of Analytical Chemistry, Faculty of Chemistry, University of Vienna, Vienna, Austria; c Joint Metabolomics Facility, University and Medical University of Vienna, Vienna, Austria; d Department of Pharmacology, Center of Physiology and Pharmacology & Comprehensive Cancer Center, Medical University of Vienna, Vienna, Austria; e Institute of Clinical Pathology, Medical University of Vienna, Vienna, Austria; f Institute of Animal Breeding and Genetics, University of Veterinary Medicine Vienna, Vienna, Austria

**Keywords:** colitis-associated colorectal cancer, azoxymethane AOM, dextran sulfate salt DSS, indoleamine 2, 3-dioxygenase 1 Ido1, interferon gamma

## Abstract

Janus kinase Tyk2 is implicated in cancer immune surveillance, but its role in solid tumors is not well defined. We used Tyk2 knockout mice (Tyk2^Δ/Δ^) and mice with conditional deletion of Tyk2 in hematopoietic (Tyk2^ΔHem^) or intestinal epithelial cells (Tyk2^ΔIEC^) to assess their cell type-specific functions in chemically induced colorectal cancer. All Tyk2-deficient mouse models showed a higher tumor burden after AOM-DSS treatment compared to their corresponding wild-type controls (Tyk2^+/+^ and Tyk2^fl/fl^), demonstrating tumor-suppressive functions of Tyk2 in immune cells and epithelial cancer cells. However, specific deletion of Tyk2 in hematopoietic cells or in intestinal epithelial cells was insufficient to accelerate tumor progression, while deletion in both compartments promoted carcinoma formation. RNA-seq and proteomics revealed that tumors of Tyk2^Δ/Δ^ and Tyk2^ΔIEC^ mice were immunoedited in different ways with downregulated and upregulated IFNγ signatures, respectively. Accordingly, the IFNγ-regulated immune checkpoint Ido1 was downregulated in Tyk2^Δ/Δ^ and upregulated in Tyk2^ΔIEC^ tumors, although both showed reduced CD8^+^ T cell infiltration. These data suggest that Tyk2^Δ/Δ^ tumors are Ido1-independent and poorly immunoedited while Tyk2^ΔIEC^ tumors require Ido1 for immune evasion. Our study shows that Tyk2 prevents Ido1 expression in CRC cells and promotes CRC immune surveillance in the tumor stroma. Both of these Tyk2-dependent mechanisms must work together to prevent CRC progression.

## Introduction

Colorectal cancer (CRC) is the third leading cause of cancer-related death in males and females.^1^ Most patients develop sporadic CRC, but approximately 2% suffer from colitis-associated CRC. In particular, patients with inflammatory bowel diseases (IBD) such as ulcerative colitis (UC) or Crohn’s disease (CD) have a threefold increased risk of developing CRC.^2–5^ The genetic and epigenetic basis of CRC has been well studied, and accurate treatment options have reduced mortality of CRC patients over the past few decades.^1^ However, treatment of metastatic CRC is still challenging, and new therapies are needed.

The best-characterized oncogenic signaling pathways are the RAS mitogen-activated protein kinase (MAPK) and phosphatidylinositol-3-kinase (PI3K) AKT pathways.^6–9^ In recent years, the Janus kinase signal transducer and activator of transcription (Jak-Stat) signaling pathway is increasingly considered crucial for development of various cancer types.^10,11^ Jak-Stat signaling is activated by cytokines, and corresponding receptors are expressed in cancer cells and immune cells.^12,13^ Therefore, Jak-Stat signaling not only mediates cancer cell-intrinsic functions but also orchestrates immune responses in the tumor microenvironment.^14^ When cytokines bind to their cognate receptors, Jaks are activated and phosphorylate-specific tyrosine residues in the intracellular receptor domains. This creates docking sites for Stat proteins that bind to the receptor and are tyrosine-phosphorylated by Jaks. The activated Stats dimerize, translocate to the nucleus, and regulate transcription of target genes involved in cell proliferation, apoptosis, cell differentiation and immunological processes.^15^ The Jak-Stat families consist of four Jaks (Jak1, Jak2, Jak3, Tyk2) and seven Stats (Stat1, Stat2, Stat3, Stat4, Stat5a, Stat5b, Stat6).

Interferons (IFNs) activate Stat1 and Stat2 via Jak1, Jak2 and Tyk2, while members of the IL-6 family activate Stat3 via gp130-associated Jak1, Jak2 and to some extend Tyk2.^15,16^ In addition, Tyk2 is activated by type I IFNs, IL-10 and IL-12 cytokine families but not by IFNγ. Although Tyk2 can activate all Stat proteins, it cannot transmit cytokine signals itself but requires Jak1 or Jak2 as a partner.^16–18^

Jak-Stat signaling plays a dual role in carcinogenesis. Chromosomal Jak translocations leading to constitutive activation of multiple oncogenic signaling pathways have been identified in hematological malignancies.^19,20^ In addition, activating mutations in Jak2 and Stat3 have been found in leukemias and solid cancers.^21^ In contrast, Stat1 is considered a tumor suppressor as it regulates expression of pro-apoptotic and anti-proliferative genes as well as molecules of the antigen presentation machinery, which increases tumor immunogenicity.^22–24^ A dual role of Jak-Stat signaling has also been observed in CRC. An oncogenic function of epithelial IL-6-Stat3 signaling was identified in the azoxymethane–dextran sulfate salt (AOM-DSS) mouse model of colitis-associated CRC.^25^ In the sporadic Apc^Min^ CRC model, Stat3 had a tumor-suppressive function in cancer cells and prevented epithelial-to-mesenchymal transition (EMT) and invasiveness.^26^ Similarly, a tumor-suppressive function of epithelial Stat1 was found in the AOM-DSS CRC model, but only in male mice.^27^ In Apc^Min^ mice, epithelial Stat1 had an oncogenic function and promoted the formation of Ido1-expressing cancer cells that mediated immune escape.^28^

The role of Tyk2 in carcinogenesis is not well defined. Oncogenic Tyk2 mutations have been found in leukemias,^29–34^ and several cancer types such as squamous cell carcinoma and prostate cancer exhibit Tyk2 activation.^35–37^ However, loss-of-function (LOF) mutations have also been described in breast, gastric and colon cancer.^38^ In this study, we examined tumor cell-intrinsic and extrinsic functions of Tyk2 in CRC. Our data revealed tumor-suppressive functions of Tyk2 in cancer cells as well as immune cells of the tumor microenvironment.

## Results

### Tyk2 deficiency promotes CRC formation

We used Tyk2^Δ/Δ^ mice with a Tyk2 germline deletion, Tyk2^ΔIEC^ mice (Tyk2^fl/fl^ Villin-cre) with specific deletion of Tyk2 in the intestinal epithelium and Tyk2^ΔHem^ mice (Tyk2^fl/fl^ Vav-cre) with specific deletion of Tyk2 in hematopoietic cells to study cancer cell-intrinsic and extrinsic functions of Tyk2 in CRC. We confirmed tissue-specific deletion of Tyk2 in these mouse strains by PCR analysis of DNA isolated from different organs and IECs (Supplementary Figure 1a). Tyk2 was completely deleted from the IECs of Tyk2^ΔIEC^ mice. Weak deletion was observed in the kidney, which is consistent with published results.^26^ Tyk2 was also completely deleted in the spleen of Tyk2^ΔHem^ mice, which consists mainly of hematopoietic cells, and partially deleted in other organs, most likely due to the presence of immune cells. As expected, deletion was complete in all organs of Tyk2^Δ/Δ^ mice. Different staining protocols were employed in Tyk2^ΔIEC^ and Tyk2^Δ/Δ^ mice to investigate whether epithelial or complete deletion of Tyk2 affects expression of Stat1 and Stat3 proteins as well as colonic homeostasis. Immunohistochemistry (IHC) showed unaltered numbers of Stat1^+^ colonic epithelial cells and lamina propria cells in Tyk2^ΔIEC^ and Tyk2^Δ/Δ^ mice (Supplementary Figure 1b,c). Stat3^+^ colonic epithelial cells and lamina propria cells were increased in Tyk2^Δ/Δ^ but not in Tyk2^ΔIEC^ mice (Supplementary Figure 1d,e). No marked activation of Stat1 and Stat3 could be observed in both cell compartments (Supplementary Figure 1b-e). Proliferating cells in the crypts and enteroendocrine cells were unchanged (Supplementary Figure 1f, g), while the number of goblet cells was slightly increased in Tyk2^Δ/Δ^ mice (Supplementary Figure 1f). These data show that specific deletion of Tyk2 in IECs has no effect on Stat1 and Stat3 expression and activation and does not affect secretory cell differentiation and crypt proliferation in the colon. However, complete deletion of Tyk2 increased Stat3 expression and promoted goblet cell differentiation to some extent.

**Figure 1.**
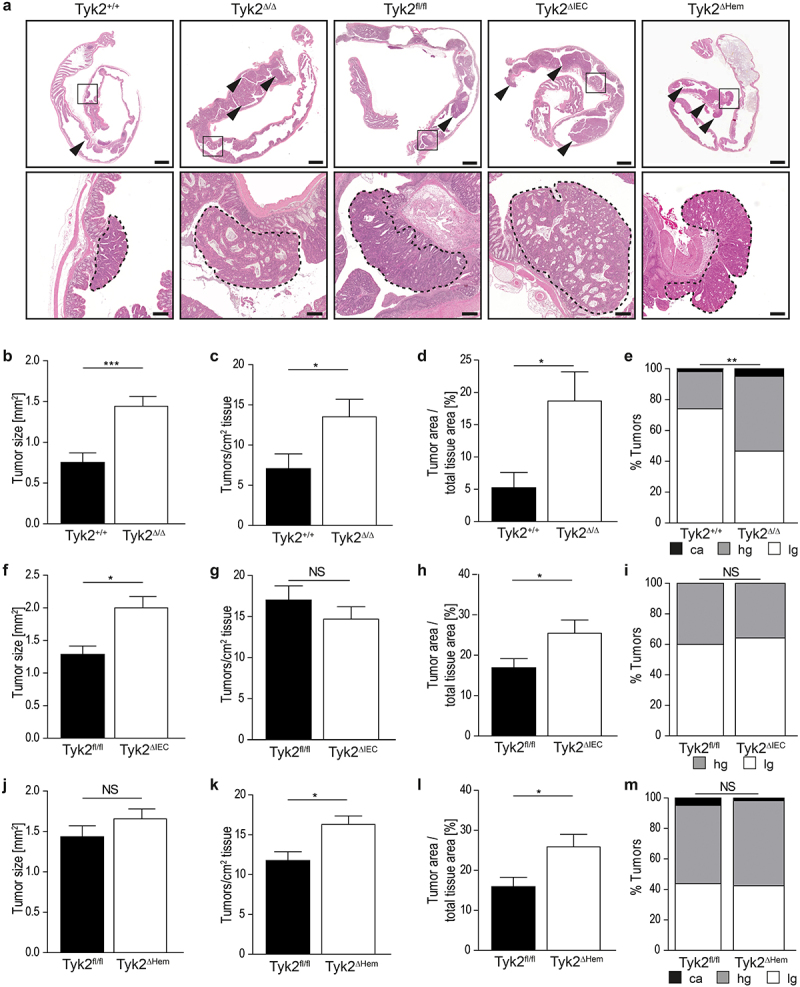
Increased colonic tumor burden in AOM-DSS-treated Tyk2^Δ/Δ^, Tyk2^ΔIEC^ and Tyk2^ΔHem^ mice. (a) H/E-stained colons from Tyk2^+/+^, Tyk2^Δ/Δ^, Tyk2^fl/fl^, Tyk2^ΔIEC^ and Tyk2^ΔHem^ mice demonstrating the increased number of colonic tumors (arrowheads) after AOM-DSS treatment in Tyk2-deficient mice. Scale bars indicate 2000 µm in the overview and 200 µm in the zoomed in portion. (b–e) Evaluation of tumor parameters and tumor grading in AOM-DSS treated Tyk2^Δ/Δ^ mice. Quantification of tumor size (*n* ≥ 54 tumors per genotype) (b), tumor multiplicity (*n* ≥ 7 mice per genotype) (c) and tumor load (*n* ≥ 7 mice per genotype) (d) in Tyk2^Δ/Δ^ compared to littermate controls. Tumor grading of colonic tumors in AOM-DSS treated Tyk2^Δ/Δ^ (*n* ≥ 54 tumors per genotype) (e). (f-i) Evaluation of tumor parameters and tumor grading in AOM-DSS treated Tyk2^ΔIEC^ mice. Quantification of tumor size (*n* ≥ 278 tumors per genotype) (f), tumor multiplicity (*n* ≥ 18 mice per genotype) (g) and tumor load (*n* ≥ 18 mice per genotype) (h) in Tyk2^ΔIEC^ compared to littermate controls. Tumor grading of colonic tumors in AOM-DSS treated Tyk2^ΔIEC^ (*n* ≥ 278 tumors per genotype) (i). (j–m) Evaluation of tumor parameters and tumor grading in AOM-DSS treated Tyk2^ΔHem^ mice. Quantification of tumor size (*n* ≥ 84 tumors per genotype) (j), tumor multiplicity (*n* ≥ 8 mice per genotype) (k) and tumor load (*n* ≥ 8 mice per genotype) (l) in Tyk2^ΔHem^ compared to littermate controls. Tumor grading of colonic tumors in AOM-DSS-treated Tyk2^ΔHem^ (*n* ≥ 84 tumors per genotype) (m). Graphs depicting the tumor size (b, f, j) are based on data from individual tumors. Tumor load (d, h, i) was calculated as tumor area per total tissue area in %. ca: carcinoma, hg: high grad adenoma, lg: low grade adenoma. Bars represent mean ± SEM. Statistical tests: Mann–Whitney test for tumor size, unpaired *t*-test for tumor multiplicity and tumor load, X^2^-test for tumor grading. NS: not significant, *p < .05, **p < .01, ***p < .001.

To investigate the role of Tyk2 in the development of CRC, we applied the AOM – DSS protocol for colitis-associated CRC to Tyk2^ΔIEC^, Tyk2^ΔHem^ and Tyk2^Δ/Δ^ mice. Macroscopic and histologic analysis of formalin-fixed colon tissues (Figure 1a) revealed increased tumor burden in all three Tyk2-deficient mouse strains (Figure 1d, h, l). Tyk2^ΔIEC^ mice developed larger tumors, whereas tumor multiplicity was unchanged (Figure 1f, g). In contrast, Tyk2^ΔHem^ mice developed more tumors, but the size was unchanged (Figure 1j, k). In Tyk2^Δ/Δ^ mice, both multiplicity and size were increased (Figure 1b, c). Furthermore, tumor progression was analyzed according to several histopathologic criteria (Supplementary Figure 2a). Increased numbers of high-grade adenomas and carcinomas were found in Tyk2^Δ/Δ^ mice but not in Tyk2^ΔIEC^ or Tyk2^ΔHem^ mice (Figure 1e,i,m). To investigate whether deletion of Tyk2 in epithelial cells has an impact on the severity of chronic colitis, we monitored the body weights of Tyk2^fl/fl^ and Tyk2^ΔIEC^ mice during the course of AOM-DSS treatment. We found a trend of increased weight loss in Tyk2^ΔIEC^ mice after the first cycle of DSS, while weight loss decreased after the second cycle, indicating that there is no difference in overall colitis severity (Supplementary Figure 2b). These data demonstrate that Tyk2 is a tumor suppressor in both cancer cell and immune cell compartments of CRC. Combined Tyk2 deletion in cancer cells and cells of the tumor stroma promotes CRC progression to invasive carcinomas.

### Differential interferon signatures in Tyk2^ΔIEC^ and Tyk2^Δ/Δ^ tumors

**Figure 2.**
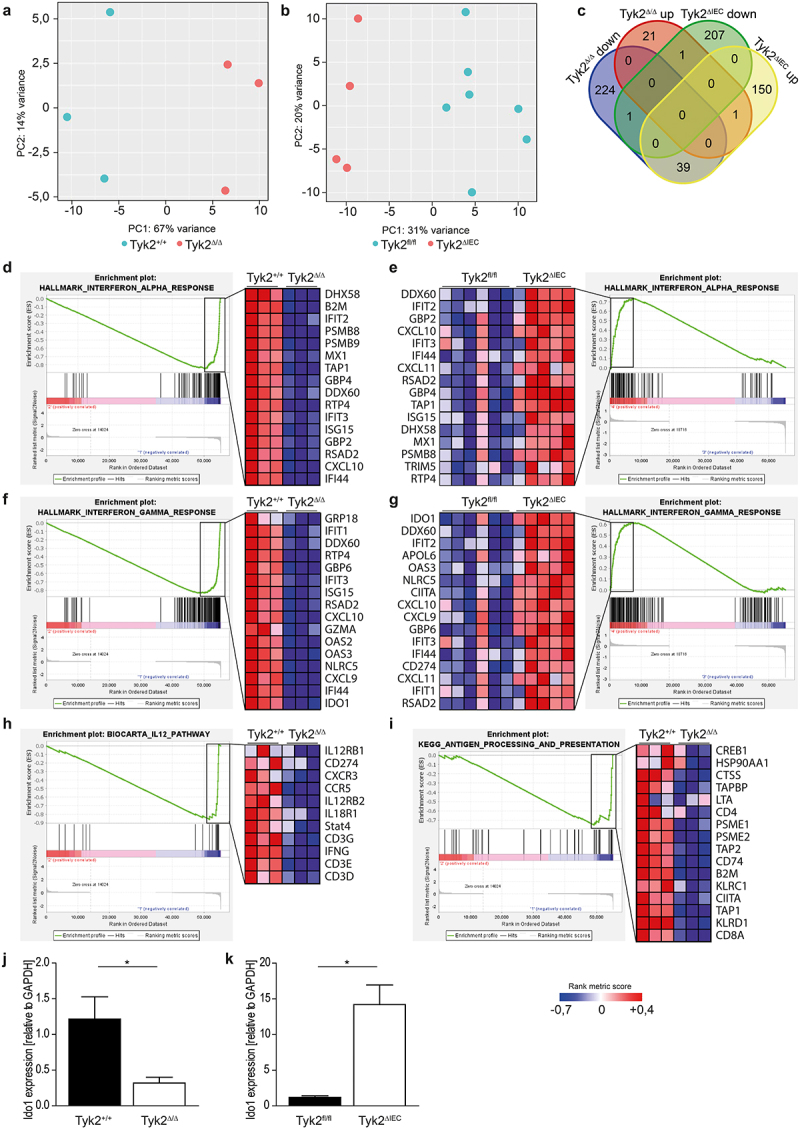
Opposing effects of epithelial and stromal Tyk2 deficiencies on IFNγ signaling. (a, b) Principal component analysis of AOM-DSS induced tumors in Tyk2^+/+^, Tyk2^Δ/Δ^ (a)Tyk2^fl/fl^ and Tyk2^ΔIEC^ (b), mice showing that tumors from Tyk2^Δ/Δ^ and Tyk2^ΔIEC^ mice are distinctly different from their respective controls. (c) Venn diagram of differentially expressed genes in Tyk2^Δ/Δ^ and Tyk2^ΔIEC^ tumors depicting an overlap of 39 genes between downregulated genes in AOM-DSS induced tumors of Tyk2^Δ/Δ^ mice and downregulated genes of tumors of Tyk2^ΔIEC^ mice. (d–i) Gene set enrichment analysis of the “Interferon Alpha Response” (d, e), “Interferon Gamma Response” (f, g), “IL12 pathway” (h) and “Antigen Processing and Presentation” (i) gene sets in Tyk2^Δ/Δ^ (d, f, h, i) and Tyk2^ΔIEC^ (e, g) tumors showing the leading edge of the enriched genes. (j, k) qPCR analysis of Ido1 expression in Tyk2^Δ^ (j) and Tyk2^ΔIEC^ (k) tumors compared to Tyk2^+/+^ and Tyk2^fl/fl^ respectively. Tyk2^+/+^ and Tyk2^Δ/Δ^: *n* = 3 pooled tumor samples per genotype, Tyk2^fl/fl^ and Tyk2^ΔIEC^: *n* ≥ 4 pooled tumor samples per genotype. Bars represent mean ± SEM. Statistical test: unpaired *t*-test. *p < .05.

Our data indicate additive tumor-suppressive functions of Tyk2 in epithelial cancer cells and stromal cells. To elucidate these functions, we performed RNA-seq analysis with tumor RNA from Tyk2^ΔIEC^ and Tyk2^Δ/Δ^ mice. Principal component analyses of RNA-seq data showed that tumors from Tyk2^Δ/Δ^ and Tyk2^ΔIEC^ mice differed from their Tyk2^fl/fl^ and Tyk2^+/+^ littermate controls (Figure 2a,b). We found 288 genes in Tyk2^Δ/Δ^ tumors and 399 genes in Tyk2^ΔIEC^ tumors that were differentially expressed at least twofold when compared to corresponding controls (Supplementary Tables 1–4). Surprisingly, Tyk2 was not among the top hits of differentially expressed genes. The floxed sequence in the targeted Tyk2 allele includes exon 3, which contains the start codon for Tyk2 protein translation. We reasoned Tyk2 deletion still results in RNA transcripts that do not yield protein but generate signals during RNA-seq analysis. Therefore, we analyzed the RNA-seq data with the Integrated Genome Viewer software (Supplementary Figure 3a). This analysis revealed a significant number of aligned reads in Tyk2 genomic regions of Tyk2^Δ/Δ^ and Tyk2^ΔIEC^ tumors. However, exon 3 reads were completely absent in Tyk2^Δ/Δ^ tumors and most Tyk2^ΔIEC^ tumors. Quantitative PCR (qPCR) analysis for Tyk2 mRNA expression using primers in exon 3 confirmed these data and showed absent and greatly reduced expression in Tyk2^Δ/Δ^ and Tyk2^ΔIEC^ tumors, respectively (Supplementary Figure 3b).

Since tumor burden was increased in Tyk2^Δ/Δ^ and Tyk2^ΔIEC^ mice, we expected similar sets of differentially expressed genes in Tyk2^Δ/Δ^ and Tyk2^ΔIEC^ tumors. However, little overlap was observed with Ccl7 as the only commonly downregulated and Pkd1l1 as the only commonly upregulated gene (Figure 2c, Supplementary Figure 3c–e). Pde11a was upregulated in Tyk2^Δ/Δ^ and downregulated in Tyk2^ΔIEC^ tumors. Thirty-nine genes were downregulated in Tyk2^Δ/Δ^ and upregulated in Tyk2^ΔIEC^ tumors. Most of these genes respond to interferon (IFN) signaling. They include members of the antigen processing and presentation machinery (Tap1), several type I interferon-stimulated genes (Ifit1-3, Ifi44, Oas3), H-2 major histocompatibility complex class I antigens (H2-Q5/Q7/Q9/T10), the immune checkpoint PD-L1 as well as genes involved in metabolic processes (Apol6, Apol9, Apol10, Ido1) (Supplementary Figure 3c-e). Using the Gene Set Enrichment Analysis tool of the Broad Institute,^39,40^ we found that gene sets correlating with IFNα and IFNγ responses were negatively enriched in Tyk2^Δ/Δ^ tumors and positively enriched in Tyk2^ΔIEC^ tumors (Figure 2d-g). IFNγ production is mainly regulated by IL12. Consequently, the gene set for IL12 signaling containing IFNγ was also negatively enriched in Tyk2^Δ/Δ^ tumors (Figure 2h) but not in Tyk2^ΔIEC^ tumors (Supplementary Tables 3 and 4). Similarly, the IFNγ-dependent gene signature for antigen processing and presentation was negatively enriched in Tyk2^Δ/Δ^ tumors (Figure 2i) but not in Tyk2^ΔIEC^ tumors (Supplementary Tables 3 and 4). Deregulation of key genes identified in the RNA-seq analysis was confirmed by qPCR (Supplementary Figure 3f, g). These data suggest that IFN-mediated immune functions are severely impaired in Tyk2^Δ/Δ^ tumors, while Tyk2^ΔIEC^ tumors exhibit enhanced IFN signatures. Therefore, the increased tumor burden in Tyk2^Δ/Δ^ and Tyk2^ΔIEC^ mice is due to different molecular mechanisms.

### Increased Ido1 protein expression in Tyk2^ΔIEC^ tumors

**Figure 3.**
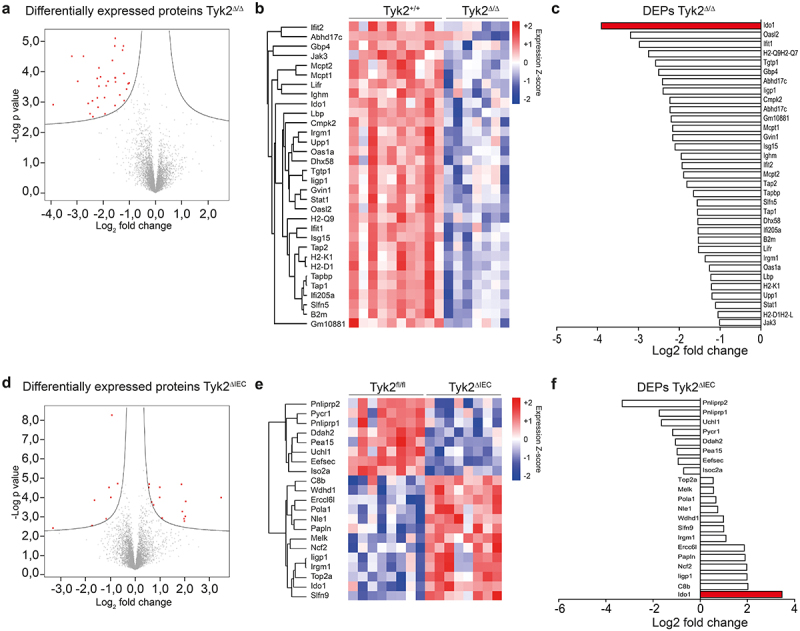
Opposing effects of epithelial and stromal Tyk2 deficiencies on Ido1 protein expression. (a-f) Analysis of differentially expressed proteins (DEPs) in AOM-DSS induced colonic tumors of Tyk2^Δ/Δ^ and Tyk2^ΔIEC^ mice compared to their respective controls. Vulcano plot (a), heatmap (b) and relative quantification (c) of DEPs in AOM-DSS induced colonic tumors of Tyk2^Δ/Δ^ mice (*n* ≥ 7 tumors per genotype). Vulcano plot (d), heatmap (e) and relative quantification (f) of DEPs in AOM-DSS induced colonic tumors of Tyk2^ΔIEC^ mice (*n* ≥ 7 tumors per genotype).

In particular, the differentially regulated Ido1 gene caught our attention in the RNA-seq dataset because it encodes for the enzyme indolamin-2,3-dioxygenase, which has immune-suppressive functions in various cancer types including CRC.^41–43^ As expected from the enrichment plots for IFNγ signaling, Ido1 mRNA expression was downregulated in Tyk2^Δ/Δ^ tumors and upregulated in Tyk2^ΔIEC^ tumors (Figure 2f,g, Supplementary Figure 3c-e). We confirmed this finding by qPCR analysis (Figure 2j,k). Furthermore, qPCR showed that Ido1 was downregulated in Tyk2^ΔHem^ tumors similar to Tyk2^Δ/Δ^ tumors (Supplementary Figure 3h). However, it is not clear whether these changes RNA expression are reflected by differential protein expression. Therefore, proteomics from Tyk2^Δ/Δ^ and Tyk2^ΔIEC^ tumor tissues was performed. Thirty-three downregulated proteins were identified in Tyk2^Δ/Δ^ tumors compared to Tyk2^+/+^ tumors that are associated with antigen processing and presentation, IFN signaling and immune responses (Figure 3a-c). The corresponding genes of most of these proteins were also differentially expressed (Figure 2, Supplementary Figure 3c-e, Supplementary Tables 1 and 2). In Tyk2^ΔIEC^ tumors, 8 downregulated and 13 upregulated proteins were identified when compared to Tyk2^fl/fl^ tumors (Figure 3d-f). While downregulated proteins were mainly implicated in protein turnover and metabolic processes, upregulated proteins were involved in DNA replication, cell cycle regulation, and extracellular matrix organization. Most genes encoding these proteins showed less than two-fold deregulation in the RNA-seq dataset, indicating post-transcriptional regulation (Supplementary Tables 3 and 4). Similarly, only a few IFNγ-regulated genes identified by RNA-seq (Figure 2e) were also significantly deregulated at the protein level in Tyk2^ΔIEC^ tumors. Importantly, the Ido1 protein was the top hit in both Tyk2^Δ/Δ^ and Tyk2^ΔIEC^ tumors. Consistent with the RNA-seq data, the Ido1 protein was downregulated in Tyk2^Δ/Δ^ tumors and upregulated in Tyk2^ΔIEC^ tumors (Figure 3a-f). These data indicate that Ido1-dependent immune suppression is reduced in Tyk2^Δ/Δ^ tumors but enhanced in Tyk2^ΔIEC^ tumors.

### Increased Ido1-expressing Paneth-like cancer cells in Tyk2^ΔIEC^ tumors

**Figure 4.**
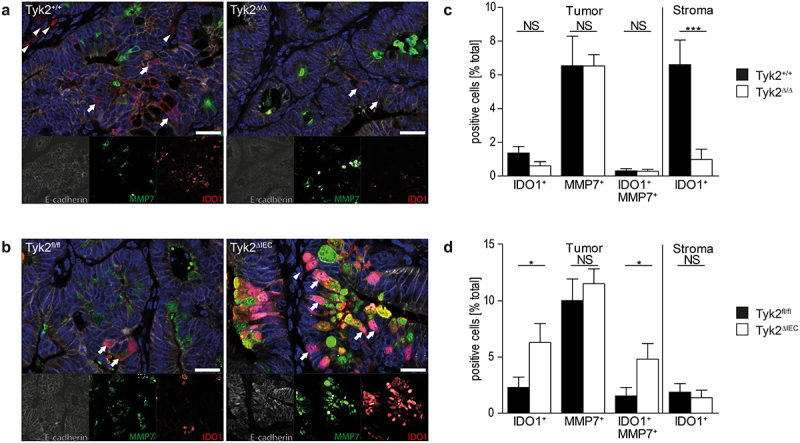
Increased numbers of Ido1 by Paneth-like cells in AOM-DSS-induced colonic tumors of Tyk2^ΔIEC^ mice. (a–d) Investigation of IDO1 expression and activity in AOM-DSS-induced colonic tumors of Tyk2^Δ/Δ^ and Tyk2^ΔIEC^ mice. IF stainings (a, b) and quantification (c, d) of IDO1-expressing tumor cells (arrows) and stromal cells (arrowheads) in colonic tumors of Tyk2^+/+^ and Tyk2^Δ/Δ^ (*n* ≥ 11 tumors in *n* = 3 animals per genotype) (a, c) and Tyk2^fl/fl^ and Tyk2^ΔIEC^ (*n* ≥ 10 tumors in *n* = 3 animals per genotype) (b, d) mice; Scale bars represent 20 µm. Bars represent mean ± SEM. NS: not significant. Statistical test: unpaired *t*-test. *p < .05, ***p < .001.

Ido1-derived metabolites promote immunosuppression and T cell anergy.^43^ We recently showed in intestinal tumors of Apc^Min^ mice that Ido1 is predominantly expressed by Mmp7^+^ Paneth-like cancer cells in an IFNγ/Stat1-dependent manner.^28^ Therefore, we performed immunofluorescence (IF) staining for Mmp7 and Ido1 in Tyk2^Δ/Δ^ and Tyk2^ΔIEC^ tumors (Figure 4a,b). Mmp7^+^ Paneth-like cancer cells were present in Tyk2^Δ/Δ^, but they did not express Ido1. Furthermore, Ido1 expression was blunted in stromal cells of Tyk2^Δ/Δ^ tumors (Figure 4a,c). In contrast, Tyk2^ΔIEC^ tumors contained an increased number of Ido1-expressing cancer cells when compared to Tyk2^fl/fl^ controls. Most of these cells were also positive for the Paneth cell marker Mmp7. Stromal Ido1 expression was unaltered in Tyk2^ΔIEC^ tumors (Figure 4b,d). These data indicate that Tyk2 impairs Ido1 expression in Paneth-like cancer cells of CRC, which is induced by stromal signals.

### Reduced antitumor immune cell infiltration in Tyk2^ΔIEC^ and Tyk2^Δ/Δ^ tumors

The presence of Ido1-expressing Paneth-like cancer cells was associated with increased tumor burden and reduced T-cell infiltration in tumors of Apc^Min^ mice, while no correlation with tumor cell proliferation or apoptosis was observed.^28^ We expected a similar phenotype in Tyk2^ΔIEC^ tumors and characterized tumor parameters, Stat expression, and immune cell infiltration by IHC and FACS analyses. IHC analysis revealed similar numbers of proliferating BrdU^+^ and apoptotic cleaved caspase3^+^ tumor cells in Tyk2^Δ/Δ^ and Tyk2^ΔIEC^ tumors when compared to Tyk2^+/+^ and Tyk2^fl/fl^ controls (Supplementary Figure 4a-d). Stat1 expression and activation were reduced in cancer cell and stromal cell compartments of Tyk2^Δ/Δ^ tumors, respectively (Supplementary Figure 4e, g), whereas Stat3 expression and activation remained unchanged (Supplementary Figure 4i, k). In contrast, expression of Stat1 was increased in cancer cells of Tyk2^ΔIEC^ tumors (Supplementary Figure 4f) while expression of Stat3 and activation of Stat1 and Stat3 were not altered (Supplementary Figure 4h, j, l).

**Figure 5.**
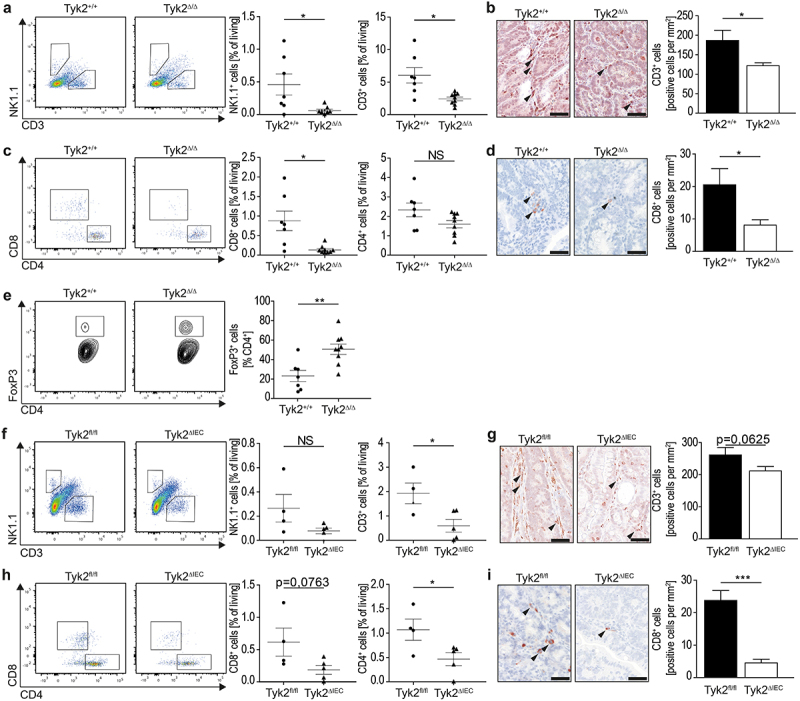
Analysis of immune cell infiltration in the tumor stroma of AOM-DSS-induced colonic tumors of Tyk2^Δ/Δ^ and Tyk2^ΔIEC^mice . (a–h) Analysis of immune cell infiltration in AOM-DSS induced colonic tumors in Tyk2^Δ/Δ^ (a–e) and Tyk2^ΔIEC^ (f-i) mice using flow cytometry and IHC. FACS plots and quantification of NK1.1^+^ NK cell and CD3^+^ Lymphocyte infiltration (*n* ≥ 4 animals per genotype, tumors from each mouse were pooled) (a, f). IHC staining and quantification of CD3^+^ Lymphocytes (arrowheads) (*n* ≥ 20 tumors in *n* ≥ 4 animals per genotype), Scale bars indicate 50 µm (b, g). FACS plots and quantification of CD4^+^- and CD8^+^ T-Lymphocyte infiltration (*n* ≥ 4 animals per genotype, tumors from each mouse were pooled) (c, h). IHC staining and quantification of CD8^+^ T-Lymphocytes (arrowheads) (*n* ≥ 29 tumors in *n* ≥ 3 animals per genotype), Scale bars indicate 50 µm (d, i). FACS plots and quantification of CD4^+^FoxP3^+^ T-Lymphocyte infiltration (*n* ≥ 7 animals per genotype, tumors from each mouse were pooled) (e). Bars represent mean ± SEM. Statistical tests: unpaired *t*-test for Tyk2^Δ/Δ^ data and Tyk2^ΔIEC^ IHC data, Mann–Whitney test for Tyk2^ΔIEC^ FACS data. NS: not significant, *p < .05, **p < .01, ***p < .001.

We next assessed immune cell infiltration into Tyk2^Δ/Δ^ and Tyk2^ΔIEC^ tumors. The extent of stromalization and the number of CD45^+^ cells in the tumor stroma were not altered in Tyk2^Δ/Δ^ and Tyk2^ΔIEC^ tumors (Supplementary Figure 5a-c, e, j). However, IHC and FACS analysis revealed reduced infiltration of NK cells and CD3^+^ T cells (Figure 5a,b, Supplementary Figure 5c,f,g) as well as CD8^+^ T cells in Tyk2^Δ/Δ^ tumors, while infiltration of CD4^+^ T cells was unchanged (Figure 5c,d, Supplementary Figure 5c, h, i) and Tregs were increased (Figure 5e, Supplementary Figure 5d). In Tyk2^ΔIEC^ tumors, infiltration of NK1.1^+^ NK cells and Tregs (Tyk2^+/+^: 1,830 ± 0,7238, *n* = 4; Tyk2^Δ/Δ^: 1,616 ± 0,3618, *n* = 5) was unchanged but CD3^+^, CD4^+^ and CD8^+^ T cells were reduced (Figure 5f-i, Supplementary Figure 5c, k–n). Additional FACS analysis for PD-1^+^ T cell populations showed a trend of CD8^+^ T cell exhaustion in Tyk2^Δ/Δ^ and Tyk2^ΔIEC^ tumors, but this was not significant (Supplementary Figure 5c, o, p). The data demonstrate that CD3^+^ and CD8^+^ T cell infiltration is impaired in Tyk2^Δ/Δ^ and Tyk2^ΔIEC^ tumors, which may reduce cancer immune surveillance and lead to increased tumor load. In contrast to Tyk2^Δ/Δ^ and Tyk2^ΔIEC^ tumors, CD3^+^ and CD8^+^ T cell infiltration was not impaired in Tyk2^ΔHem^ tumors. However, FoxP3^+^ cells were increased while Granzyme B^+^ cells were decreased (Supplementary Figure 6a–e), which may also reduce cancer immune surveillance and lead to increased tumor multiplicity. The data from Tyk2^ΔHem^ tumors suggest that deletion of Tyk2 in cancer cells is required for reduced CD8^+^ T cell infiltration.

## Discussion

The role of Tyk2 in solid tumors is not well defined and oncogenic as well as tumor-suppressive functions have been reported. Here, we show that Tyk2 is a tumor suppressor in CRC. Using three different mouse models with Tyk2 deletion and the AOM-DSS protocol for colitis-associated CRC, we demonstrate additive tumor-suppressive functions of Tyk2 in cancer cells and in the immune microenvironment. Tyk2 deletion in cancer cells or in immune cells increased the tumor burden, but only combined deletion in both cellular compartments promoted tumor progression. The qualitative differences in tumor formation in the three mouse models could be due to differential infiltration and activation of immune cells modulated by loss of Tyk2 in both the hematopoietic system and the epithelial cancer cell compartment. Our data also suggest that the use of Tyk2 inhibitors to treat IBD, which is currently being investigated in clinical trials, should be viewed with caution as it blocks Tyk2 in all cell types and could promote progression of as yet unrecognized neoplastic lesions.

There is ample evidence that Tyk2 is involved in immune surveillance of cancer.^44–46^ Tyk2 is required for IL12 production by myeloid cells, which triggers a Th1 response with enhanced IFNγ production and tumor cell killing by CD8^+^ T cells and NK cells.^47^ Therefore, we expected similar cancer phenotypes in Tyk2^Δ/Δ^ and Tyk2^ΔHem^ mice since both mouse models lack Tyk2 in immune cells. However, Tyk2^Δ/Δ^ mice developed significantly larger and more aggressive tumors, while the increase in tumor burden in Tyk2^ΔHem^ mice was rather subtle. Only tumor multiplicity was increased, suggesting that hematopoietic Tyk2 is involved in early immune surveillance of CRC but not in tumor progression. Interestingly, Tyk2^Δ/Δ^ and Tyk2^ΔIEC^ tumors showed a similar reduction of CD8^+^ T cell infiltration that was not found in Tyk2^ΔHem^ tumors. This indicates that Tyk2 deletion in the epithelial cancer cell compartment is responsible for reduced infiltration of CD8^+^ T cells into tumors.

While the importance of Tyk2 in cancer immune surveillance is well established, there is little evidence of Tyk2 functions in cancer cells.^46^ Tyk2 has been shown to drive human cancer cell invasion and metastasis in a cancer cell-intrinsic manner.^14^ The AOM-DSS model does not allow assessment of metastasis, but comparison of tumor data in Tyk2^Δ/Δ^ and Tyk2^ΔIEC^ mice rather suggest an anti-metastatic function of Tyk2 in cancer cells, which becomes evident when Tyk2-mediated immune surveillance is impaired. We recently showed that Tyk2 mediates protective IL22 signaling in gut epithelial cells during DSS-induced acute colitis.^48^ We did not observe increased lethality of Tyk2^Δ/Δ^, Tyk2^ΔHem^ or Tyk2^ΔIEC^ mice during DSS-induced induction of chronic colitis in the AOM-DSS protocol. Furthermore, gene expression data provided no evidence of impaired IL22 signaling and tumor proliferation rates were unaffected, suggesting that cancer phenotypes are IL22-independent.

The RNA-seq and proteomics data revealed different molecular profiles of Tyk2^Δ/Δ^ and Tyk2^ΔIEC^ tumors. We found deregulated proteins also in the RNA-seq dataset, but most candidates were less than two-fold deregulated at the transcriptional level. Therefore, we hypothesize that post-transcriptional processes are strongly involved in the deregulation of proteins. Consistently, Tyk2 has previously been shown to be involved in the regulation of translation.^49^ Most differentially expressed genes and proteins were part of an IFN signature and were regulated in opposite directions in Tyk2^Δ/Δ^ and Tyk2^ΔIEC^ tumors suggesting differential cancer genesis and immunoediting. However, it remains unclear how cancer cell-specific Tyk2 deletion can lead to increased IFN signaling as seen in Tyk2^ΔIEC^ tumors. Although IFNγ plays a key role in cancer immune surveillance, sustained activity can result in upregulation of immune checkpoints that promote immune evasion.^50^ We found strong upregulation of the IFNγ-regulated immune checkpoint Ido1 in Tyk2^ΔIEC^ tumors. The role of Ido1 in CRC and in cancer in general is not well defined, but several studies have reported an oncogenic activity. Kras^G12D^-induced lung carcinogenesis and lung metastasis of orthotopically transplanted 4T1 mouse mammary cancer cells was reduced in Ido1 knockout mice.^51^ In addition, Ido1 knockout mice were resistant to skin cancer formation after DMBA/PMA treatment. In the DMBA/PMA model, Ido1 expression was induced by skin inflammation in a specific subset of plasmacytoid dendritic cells in the draining lymph nodes, which promoted immunosuppression and skin cancer formation.^52^ In contrast to lung and skin carcinogenesis, formation of colitis-associated CRC was increased in Ido1 knockout mice after treatment with dimethylhydrazine and DSS, indicating a tumor-suppressive role.^53^ However, a recent study showed that specific deletion of Ido1 in intestinal epithelial cells reduced AOM-DSS-induced colitis-associated CRC formation in mice.^54^ These data revealed cell-type specific tumor promoting and tumor suppressing activities of Ido1 in colitis-associated CRC. We recently identified Ido1-expressing Paneth-like cancer cells in tumors of Apc^Min^ mice that prevent T cell infiltration and promote immune evasion. Ido-1 expression by these cells depends on Stat1 and IFNγ signaling.^28^ Tyk2^ΔIEC^ tumors expressed higher levels of Stat1 and contained increased numbers of Ido1-expressing Paneth-like cancer cells. These cells may be responsible for reduced T cell infiltration in Tyk2^ΔIEC^ tumors and allow tumor growth despite enhanced IFNγ signaling and intact immune surveillance. In contrast, IL12 and IFNγ signaling was attenuated in Tyk2^Δ/Δ^ tumors, resulting in impaired cancer immune surveillance with reduced NK cell and T cell infiltration. Ido1-expressing Paneth-like cancer cells were almost absent in Tyk2^Δ/Δ^ tumors and expression of antigen presentation machinery genes was reduced. This indicates that tumors in Tyk2^Δ/Δ^ are poorly immunoedited and can progress to aggressive cancers without developing distinct immune evasion mechanisms.

Ido1 is the rate-limiting enzyme of the kynurenine pathway. It catalyzes oxidation of tryptophan to N-formylkynurenine, which is further converted to kynurenine and several kynurenine metabolites.^55^ High Ido1 activity leads to tryptophan depletion and a corresponding increase of kynurenine metabolites. Tryptophan depletion negatively affects proliferation of effector T cells, while kynurenine promotes Treg differentiation via the aryl hydrocarbon receptor.^56,57^ Consequently, Ido1 creates an immunosuppressive microenvironment with reduced CD8^+^ T cell count and expansion of Tregs.^58–60^ Ido1 is frequently overexpressed in human CRC, which is associated with reduced levels of serum tryptophan and increased levels of kynurenine metabolites.^61–63^ In tumors, Ido1 is expressed by infiltrating myeloid cells and neoplastic epithelial cells.^42,43,64^ We have shown that the neoplastic epithelium of CRC is an important source of kynurenine.^28^ Similarly, Ido1-expressing neoplastic epithelial cells of pancreatic ductal adenocarcinomas (PDACs) were critically involved in immune escape.^65^ We have not performed metabolomics of mouse Tyk2-deficient CRCs. Ido1 expression was reduced in stromal immune cells but not in neoplastic cells of Tyk2^Δ/Δ^ tumors. In contrast, Ido1 expression was unaffected in stromal immune cells but increased in neoplastic cells of Tyk2^ΔIEC^ tumors. Therefore, metabolomics of Tyk2^Δ/Δ^ and Tyk2^ΔIEC^ would help to identify the main source of kynurenine metabolites in CRC.

The expression of several B cell markers such as CD19, CD20 and Pax5 was downregulated in Tyk2^Δ/Δ^ tumors. This suggests a lack of antigen-specific B cells, which would impair FC-mediated phagocytosis and antibody-dependent cellular cytotoxicity by NK cells. Alternatively, a previously identified cell population with dendritic cell characteristics but expressing the B cell markers CD19 and Pax5 could be reduced in Tyk2^Δ/Δ^ tumors. These B lymphoid-like cells are a major source of Ido1 in some tumors and have immunosuppressive activity on T cells.^66^ Interestingly, the Ido1 pathway could also prevent complement deposition in tumors.^55^ It has been shown that pharmacological or genetic Ido1 inhibition combined with chemo-radiotherapy resulted in extensive complement deposition in mouse brain tumors.^67^ It is therefore possible that complement deposition is reduced in Tyk2^Δ/Δ^ tumors. Furthermore, expression of complement receptor 2, which is present on B cells and follicular dendritic cells, was also greatly reduced in Tyk2^Δ/Δ^ tumors, which could further impair complement-mediated anticancer immunity. In conclusion, we have identified a tumor-suppressive function of Tyk2 in stromal and CRC cells. Tyk2 blocks induction of the IFNγ-regulated immune checkpoint Ido1 in Paneth-like cancer cells by an unknown mechanism. Deletion of Tyk2 in Paneth-like cancer might change Jak-Stat signaling in a cell-intrinsic manner. Stimulation with Jak-Stat-activating cytokines from the tumor microenvironment would enhance formation of Stat1-containing transcription factor complexes that lead to increased expression of type I and II interferon target genes including Ido1. The murine Ido1 promoter is not well characterized but the human Ido1 promoter contains multiple ISRE and GAS sites that respond to ISGF3 (Stat1-Stat2-IRF9) complexes and Stat1 homodimers, respectively.^68^ An increased amount of such complexes in Tyk2-deficient Paneth-like cancer cells would enhance Ido1 expression. The generation of these cells could also be promoted in early neoplastic lesions of IBD patients by treatment with Tyk2 inhibitors, which would increase the risk of immune escape.

## Materials and methods

### Mice and in vivo experiments

Tyk2^Δ/+^, Tyk2^fl/+^, Villin-cre and Vav-cre mice have been described previously.^69–71^ Tyk2^Δ/+^ mice were generated by crossing Tyk2^fl/+^ mice with a CMV-cre germline deleter strain, which was then outcrossed. Tyk2^Δ/+^ mice were crossed to produce Tyk2^Δ/Δ^ and control Tyk2^+/+^ mice. Tyk2^fl/fl^ Villin-cre and Tyk2^fl/fl^ Vav-cre mice were crossed with Tyk2^fl/fl^ mice to generate cre-negative Tyk2^fl/fl^ control mice and cre-positive Tyk2^ΔIEC^ and Tyk2^ΔHem^ mice. Mice were kept on a C57BL/6 genetic background and housed under standard conditions at the Dezentrale Biomedizinische Einrichtung of the Medical University Vienna. Autochthonous CRC development was induced using the AOM-DSS protocol.^72^ Mice were injected intraperitoneally with 12.5 mg/kg azoxymethane (AOM, Sigma, A5486) and, after 5 d of recovery, treated with two cycles of 2.5% dextrane sulfate salt (DSS, MP Biomedicals LCC, 160110) and one cycle of 2.0% DSS (w/v) via drinking water. DSS treatment lasted 5 d each with a 14-d interval of normal drinking water between cycles.^72^ After the last DSS cycle, mice recovered for 30 d before they were sacrificed. All mouse experiments were performed in accordance with Austrian and European laws and with the general regulations specified by the Good Scientific Practices guidelines of the Medical University of Vienna.

### Genotyping by polymerase chain reaction (PCR)

Genotyping of Tyk2 was performed with 5’-GCAAGCCTGGGTTACATGAG-3’ and 5’-TGGACTGGAACTTGTGAGGA-3’ primers. The Villin-Cre transgene was detected with 5’-CGGTCGATGCAACGAGTGATGAGG-3’ and 5’-CCAGAGACGGAAATCCATCGCTCG-3’ primers. Vav-Cre genotyping was performed with 5’-TCAGAGTGAAGGACATCTCCCGCACC-3’ and 5’-GTGGCAGAAGGGGCAGCCACACCATT-3’ primers.

### Histochemistry, immunohistochemistry (IHC) and immunofluorescence (IF)

The colons of AOM-DSS-treated mice were isolated and flushed with phosphate-buffer saline (PBS) and fixed with 4% formaldehyde as “swiss rolls”. Tissues were embedded in paraffin, cut into 4 µm thick sections tissue sections, deparaffinized in xylene and rehydrated in a decreasing ethanol series. Immunohistochemistry (IHC), immunofluorescence (IF) and hematoxylin & eosin (H&E) staining were performed using standard procedures.^72^ Briefly, for IHC and IF staining, antigen retrieval was performed using a pH6 citrate buffer or pH9 Tris-EDTA buffer depending on the antigen. For IHC staining, multiple blocking steps using hydrogen peroxide, avidin, biotin and the Mouse to Mouse HRP Staining System (ScyTek, MTM003-IFU) were performed. After blocking, the slides were stained using antibodies for BrdU (Abcam, ab6326), cleaved caspase 3 (Cell Signaling, 3700), Stat1 (Santa Cruz, sc-592), pStat1 (Cell Signaling, 9167), Stat3 (Santa Cruz, sc-7179), pStat3 (Cell Signaling, 9145), CD8 (Invitrogen, MA1-80231) and CD3 (Dako, A045229-2) in 1% BSA. IHC staining was detected with AEC substrate chromogen (Dako, K3464) or ImmPACT™ DAB Peroxidase Substrate Kit (VECSK-4105). Tissue sections for IF staining were blocked with 10% goat serum in PBS and incubated with primary antibodies for Ido1 (BioLegend, 654002), MMP7 (Cell Signaling, 3801) or E-cadherin (Abcam, ab11512) in 5% goat serum in PBS. The slides were incubated with fluorochrome-coupled secondary antibodies against rabbit (AF488, Fisher, A11034), mouse (Daylight649, BioLegend, 405312) or rat (Cy3, Fisher, A10522) immunoglobulins and counterstained with DAPI. For H&E staining, sections were incubated in hematoxylin for 10 min, dehydrated in an increasing ethanol series and stained with eosin for 2 min. For Alcian blue staining, the sections were incubated for 10 min each in Alcian blue and nuclear fast red. Stained “Swiss rolls” were scanned using a Pannoramic Midi Slide Scanner (3D Histech, 40× objective) and staining intensities were analyzed with Definiens™ TissueStudio™ histomorphometry software (Definiens) as described previously.^72^

### Flow Cytometry

AOM-DSS-induced colorectal tumors from mice were pooled, minced and digested in 2 mL PBS containing 2% (v/v) FCS and 1 mg/mL collagenase IV (Life Technologies, 17104–019) for 45 min at 37°C under shaking. Afterward, the cells were strained through a 70 µm mesh and washed twice with 30 ml PBS. The cells were taken up in 200 µL of cell staining buffer and split in 4 equal parts. Extracellular stainings were performed using antibodies against 7-AAD (BioLegend, 420404), CD3 (BioLegend, B100305), CD4 (BioLegend, B116011), CD8a (BioLegend, B100737), CD45 (BioLegend, B103115), PD-1 (BioLegend, B135205), NK1.1 (BioLegend, B108739). For intracellular staining cells were fixed, permeabilized using the True nuclear TF staining buffer kit (BioLegend 424401) and stained with a FoxP3 (BioLegend, 126408) antibody. The data were collected using a FACS Fortessa (BD) and analyzed with FlowJo software.

### RNA sequencing (RNA-seq)

RNA from AOM-DSS-induced macrodisected tumors was isolated using TRIzol Reagent (Thermo Fisher Scientific, 15596018) and purified using the RNeasy® Protect Mini Kit (Quiagen, 74127) according to the manufacturer’s manual. RNA-sequencing was performed at the Core Facilities of the Medical University of Vienna. Sequencing libraries were prepared using the NEBNext Poly(A) mRNA Magnetic Isolation Module and the NEBNext Ultra™ II Directional RNA Library Prep Kit for Illumina according to manufacturer’s protocols (New England Biolabs). Libraries were QC-checked on a Bioanalyzer 2100 (Agilent) using a High Sensitivity DNA Kit for correct insert size and quantified using Qubit dsDNA HS Assay (Invitrogen). Pooled libraries were sequenced on a NextSeq500 instrument (Illumina) in 1x75bp single-end sequencing mode. Approximately 25 million reads were generated per sample. Reads in fastq format were aligned to the mouse reference genome version GRCh38^73^ with Gencode mV23 annotations^74^ using STAR aligner^75^ version 2.6.1a in 2-pass mode. Reads per gene were counted by STAR, and differential gene expression was calculated using DESeq2^76^ version 1.20.0. and analyzed using the Gene Set Enrichment Analysis (GSEA) tool provided by the Broad Institute.^39,40^

### Quantitative PCR (qPCR)

RNA from tissues and cells was isolated using TRIzol Reagent (Thermo Fisher Scientific, 15596018) and reverse transcribed using the QuantiTect Reverse Transcription Kit (Qiagen, 205313). For qPCR analysis, the GoTaq® qPCR Master Mix (Promega, A6001/2) and CFX96™ Real-Time System (Bio-Rad) were used with the following mouse primers:

GAPDH: 5’-TGTTTGTGATGGGTGTG-3’, 5’-TACTTGGCAGGTTTCTC-3’; Tyk2_Exon3: 5’-CCCACAGGATGCTTGATGGT-3’, 5’-CGACTTTGTGTGCGATGTGG-3’; Ido1: 5’-ATGTGGGCTTTGCTCTACCA-3’, 5’-AAGCTGCCCGTTCTCAATCA-3’; Tap1: 5’-ACCAGCTGTCAGGAGGTCAG-3’, 5’-CTTGGGGCTCTCATACAGGA-3’; Cxcl9: 5’-CGATCCACTACAAATCCCTCA-3‘, 5‘-TAGGCAGGTTTATCTCCGT-3’; Ifi44: 5’-AGTCCTGTGAAGTCCAAGCTG-3’, 5’-CAGCTGCCACTCTGAGACAT-3’.

### Proteomics

Single AOM-DSS induced colorectal tumors from Tyk2^Δ/Δ^, Tyk2^ΔIEC^ mice and corresponding control mice were harvested, washed with PBS, snap frozen in liquid nitrogen (and stored at −80°C). Sample preparation and proteomics analysis were essentially performed as described before, with minor changes.^77^ The tissue samples were separately homogenized in 130 μl lysis buffer (8 M urea, 50 mM TEAB, 5% SDS) using the Covaris S220 homogenizer. For enzymatic digestion, a protocol using the S-trap technology was employed.^78^ Briefly, reduction and alkylation of 20 µg solubilized protein was achieved with 64 mM DTT and 48 mM IAA, respectively. After addition of trapping buffer (90%vol/vol methanol, 0.1 M triethylammonium bicarbonate), samples were loaded onto S-trap cartridges, thoroughly washed and subsequently digested using Trypsin/Lys-C Mix at 37°C for 2 h. Peptides were eluted, dried and stored at −20°C until LC-MS analyses. Dried samples were reconstituted in 5 µl of 30% FA containing 4 synthetic standard peptides and diluted with 40 µl of loading solvent (97.9% H2O, 2% ACN, 0.05% trifluoroacetic acid). LC‐MS/MS analyses were performed using a Dionex Ultimate 3000 nano LC‐system coupled to a timsTOF pro mass spectrometer (Bruker Daltonics). Five microliters of peptide solution were loaded on a 2 cm × 100 µm C18 Pepmap100 pre‐column (Thermo) at a flow rate of 10 µl/min using mobile phase A. Afterward, peptides were eluted from the pre‐column to a 25 cm × 75 µm 25 cm Aurora Series emitter column (Ionopticks) at a flow rate of 300 nL/min and separation was achieved using a gradient of 8% to 40% mobile phase B (79.9% acetonitrile, 20% H2O, 0.1% FA) over 90 mins. Mass spectrometric analyses was accomplished using the timsTOF Pro mass spectrometer (Bruker) equipped with a captive spray ion source run at 1600 V. Furthermore, the timsTOF Pro mass spectrometer was operated in the Parallel Accumulation-Serial Fragmentation (PASEF) mode. Trapped ion mobility separation was achieved by applying a 1/k0 scan range from 0.60–1.60 V.s/cm^2^ resulting in a ramp time of 100 ms. All experiments were performed with ten PASEF MS/MS scans per cycle leading to a total cycle time of 1.16s. MS and MS/MS spectra were recorded using a scan range (m/z) from 100 to 1700. Furthermore, the collision energy was ramped as a function of increasing ion mobility from 20 to 52 eV and the quadrupole isolation width was set to 2 Th for m/z < 700 and 3 Th for m/z > 700.

MaxQuant 1.6.17.0^79^ employing the Andromeda search engine was used for protein identification against the UniProt Database (version 12/2019 with 20.380 entries) allowing a mass tolerance of 20 ppm for MS spectra and 40 ppm for MS/MS spectra, an FDR <0.01 and a maximum of two missed cleavages. Furthermore, search criteria included carbamidomethylation of cysteine as fixed modification and methionine oxidation, N-terminal protein acetylation as variable modifications. Data evaluation was performed using the Perseus software V.1.6.14.0. Proteins that were detected in less than 70% of the samples were filtered out. Missing values from the remaining proteins were filled up according to a normal distribution with a width of 0.3 and a down shift of 1.8. The GOBPs were annotated using the integrated database of the Perseus software.

### Statistics

All values are given as mean ± standard error of the mean (SEM). All data distributions were tested for normality using the Kolmogorov–Smirnov test. The calculation of significant differences between two groups was performed using unpaired *t*-test (for normal distributed data) and the Mann-Whitney test (for non-normal distributed data). Significant differences in tumor grading were calculated by performing a X^2^-test. Significant differences between experimental groups were *p < .05, **p < .01 and ***p < .001.

## Supplementary Material

Supplemental Material

## Data Availability

The data that support the findings of this study are available from the corresponding author upon reasonable request [http://krebsforschung.meduniwien.ac.at/forschung/forschungsschwerpunkte/zellulaere-und-molekulare-tumorbiologie/robert-eferl/].
