# Supplementary material for: Tyk2 is a tumor suppressor in colorectal cancer
Source: Oncoimmunology. 2022 Sep 26;11(1):2127271. doi: 10.1080/2162402X.2022.2127271 (PMC9519006; doi:10.1080/2162402X.2022.2127271)
Supplement: Supplemental Material [file KONI_A_2127271_SM6198.zip › Supplementary Figure Legends.docx]

**Supplementary Figure Legends**

**Suppl. Figure 1:** Characterization of Tyk2-deficient mouse strains.
**(a)** DNA was isolated from various organs and intestinal epithelial cells of Tyk2^fl/fl^, Tyk2^ΔIEC^, Tyk2^ΔHem^, Tyk2^+/+^, and Tyk2^Δ/Δ^ mice. The conditional deletion of Tyk2 was evaluated by PCR analysis. Spl.: spleen; kid.: kidney; IEC: intestinal epithelial cells. **(b-e)** Characterization of total Stat1 and Stat3 expression and activation in intestinal epithelial cells and the lamina propria of untreated Tyk2^+/+^, Tyk2^Δ/Δ^ Tyk2^fl/fl^ and Tyk2^ΔIEC^ mice. IHC staining and quantification of Stat1, pStat1 **(b, c)**, Stat3 and pStat3 **(d, e)** positive cells. Stat1 and Stat3 positive cells are indicated with arrowheads. Stat1 and Stat3 activation in untreated mice could not be detected. (Tyk2^+/+^: 3 mice, 1 HPF per mouse; Tyk2^Δ/Δ^: 3mice, 1 HPF per mouse; Tyk2^fl/fl^: 3 mice, 1 HPF per mouse; Tyk2^ΔIEC^: 6 mice, 1 HPF per mouse). **(f, g)** Frequency of mucosal cell types in the colonic intestinal epithelium of untreated Tyk2^+/+^, Tyk2^Δ/Δ^, Tyk2^fl/fl^ and Tyk2^ΔIEC^ mice. Mucosal cell types were stained with with IHC for Synaptophysin positive enteroendocrine cells and with IHC for BrdU incorporation in proliferating cells and Alcian blue for Goblet cells in Tyk2^+/+^ and Tyk2^Δ/Δ^ **(f)** and Tyk2^fl/fl^ and Tyk2^ΔIEC^ **(g)** mice. Corresponding cell types are indicated with arrowheads. (BrdU: n ≥ 106 crypts in n ≥ 3 animals per genotype, Synaptophysin: n ≥ 48 crypts in n = 3 animals per genotype, Alcian blue: n ≥ 290 crypts in n ≥ 3 animals per genotype). Scale bars indicate 50 μm. Bars represent mean ± SEM. Statistical test: unpaired *t*-test. ND: not detected, NS: not significant,*p < 0.05, **p < 0.01, ***p < 0.001.

**Suppl. Figure 2:** Histological analysis of colonic tumors and weight curve of AOM-DSS treated mice
**(a)** Representative H&E-stained images of AOM-DSS-induced low-grade adenomas (l.g.), high-grade adenomas (h.g.) and carcinomas in Tyk2^fl/fl^, Tyk2^ΔIEC^, Tyk2^ΔHem^, Tyk2^+/+^ and Tyk2^Δ/Δ^ mice. The squares in the top low-magnification images indicate the positions of the high-magnification images below. Tumors were graded according to the following criteria. Low grade adenomas: loss or significant decrease of goblet cells, cytoplasmic hyperchromasia of nuclei which show regular basal orientation and are not markedly enlarged (arrows). High grade adenomas: back-to-back glands with no intervening stroma, total loss of polarity by atypical cells (arrows), sometimes mucin-producing cells forming lumen, technically appearing cribriform. Carcinomas: nuclei round and vesicular, tumor glands do not have a significant number of goblet cells, glands are often fused with secondary lumina and show an increasing complexity, invasion through the muscularis mucosae (arrow) into the submucosa so that the invasion front of the tumor can be seen in the submucosa and is frequently in close proximity to submucosal blood vessels, frequent presence of desmoplasia. Scale bars indicate 200µm in the 5x and 20µm in the 40x magnification images respectively. **(b)** Weight curve of Tyk2^fl/fl^ and Tyk2^ΔIEC^ mice treated with AOM-DSS. Values are % of body weight at day 0 (time of AOM injection).

**Suppl. Figure 3**: Validation of the RNA-seq analysis of AOM-DSS induced colonic tumors.
**(a)** Alignment analysis of Tyk2 in tumors from Tyk2^+/+^, Tyk2^Δ/Δ^, Tyk2^fl/fl^ and Tyk2^ΔIEC^ mice. The enhanced section shows the aligned reads in exon 3 of the Tyk2 gene. **(b)** qPCR analysis of Tyk2 expression in single tumors from Tyk2^+/+^, Tyk2^Δ/Δ^, Tyk2^fl/fl^ and Tyk2^ΔIEC^ mice. Tyk2 was not detected in Tyk2^Δ/Δ^ tumors and only detected in a single Tyk2^ΔIEC^ tumor. (Tyk2^+/+^ and Tyk2^fl/fl^: n ≥ 4 tumors; Tyk2^Δ/Δ^ and Tyk2^ΔIEC^:n ≥ 21 tumors). **(c)** List of overlapping differentially expressed genes between Tyk2^Δ/Δ^ and Tyk2^ΔIEC^ tumors. **(d, e)** Vulcano plots of differentially expressed genes in Tyk2^Δ/Δ^ **(d)** and Tyk2^ΔIEC^ **(e)** tumors showing the overlapping genes between the Tyk2^Δ/Δ^ and Tyk2^ΔIEC^ tumors. **(f, g)** Evaluation of RNA-seq results using qPCR analysis. Quantification of Tap1, Cxcl9 and Ifi44 gene expression in Tyk2^+/+^, Tyk2^Δ/Δ^ **(f)**, Tyk2^fl/fl^ and Tyk2^ΔIEC^ **(g)** mice (n≥ 5 single tumor RNAs per genotype). **(h)** qPCR analysis of Ido1 expression in Tyk2^ΔHem^ mice (n≥ 8 single tumor RNAs per genotype). Bars represent mean ± SEM. Statistical test: unpaired *t*-test. ND: not detected, *p < 0.05, **p < 0.01, ***p < 0.001.

**Suppl. Figure 4:** Immunohistological analysis of AOM-DSS induced colonic tumors
**(a-l)** IHC stainings and quantification of BrdU^+^ **(a, b)** cleaved caspase3^+^ **(c, d)**, Stat1^+^ **(e, f)**, pStat1^+^ **(g, h)**, Stat3^+^ **(i, j)** and pStat3^+^ **(k, l)**, tumor and stromal cells in AOM-DSS induced colonic tumors in Tyk2^Δ/Δ^ mice **(a, c, e, g, i, k)** and Tyk2^ΔIEC^ mice **(b, d, f, h, j, l)** compared to their respective littermate controls (n ≥ 9 tumors in n ≥3 animals per genotype). Positive tumor cells are indicated with arrowheads, positive stromal cells are indicated with arrows. Scale bars represent 50µm. Bars represent mean ± SEM. Statistical test: unpaired *t*-test. NS: not significant, *p < 0.05.

**Suppl. Figure 5:** Flow cytometric analysis of Analysis of AOM-DSS induced colonic tumors
**(a, b)** Stromalization of AOM-DSS induced colonic tumors in Tyk2^Δ/Δ^ **(b)** and Tyk2^ΔIEC^mice **(c)** compared to their respective littermate controls (n ≥ 17 tumors in n ≥3 animals per genotype). **(c, d)** Gating strategies of the flow cytometry analysis of AOM-DSS induced colonic tumors of Tyk2^+/+^, Tyk2^Δ/Δ^, Tyk2^fl/fl^ and Tyk2^ΔIEC^ mice. Gating strategy for the analysis of NK cells, CD3^+^-, CD4^+^-, CD8^+^-T cells and PD1^+^ cells in Tyk2^+/+^, Tyk2^Δ/Δ^, Tyk2^fl/fl^ and Tyk2^ΔIEC^ mice **(c)**. Gating strategy for the analysis of CD4^+^FoxP3^+^-Treg cells in Tyk2^+/+^ and Tyk2^Δ/Δ^ mice **(d)**. **(e-p)** Quantification of living CD45^+^ **(e, j)** as well as NK1.1^+^ **(f, k)**, CD3^+^**(g, l)**, CD8^+^**(h, m)**, CD4^+^**(i, n)** and CD4^+^FoxP3^+^ cell infiltration relative to living CD45^+^ cells in Tyk2^+/+^; Tyk2^Δ/Δ^ **(e-i)**, Tyk2^fl/fl^ and Tyk2^ΔIEC^ mice **(j-n)**. **(o, p)** Quantification of CD8^+^- and CD4^+^-T cell exhaustion. Medium Fluorescence Intensity (MFI) of PD1-expressing CD8^+^ and CD4^+^-T cells in Tyk2^+/+^; Tyk2^Δ/Δ^ **(o)**, Tyk2^fl/fl^ and Tyk2^ΔIEC^ mice **(p)**. n ≥ 4 animals per genotype, tumors from each mouse were pooled. Data are presented as mean ± SEM. Statistical tests: unpaired *t*-test for Tyk2^Δ/Δ^ data, Mann-Whitney test for Tyk2^ΔIEC^ data. NS: not significant, *p < 0.05.

**Suppl. Figure 6:** Immune cell infiltration in AOM-DSS induced colonic tumors in Tyk2^ΔHem^ mice
**(a-e)**. Quantification of IHC stained CD3^+^- **(a)**, CD4^+^- **(b)**, CD8^+^- **(c)**, FoxP3^+^- **(d)** and GzmB^+^- **(e)** cells in Tyk2^ΔHem^ mice. Positive cells are indicated with arrowheads. n ≥ 20 tumors in n ≥ 4 animals per genotype. Scale bars indicate 50 μm. Data are presented as mean ± SEM. Statistical tests: unpaired *t*-test. NS: not significant, *p < 0.05, ***p<0,001.
