# Supplementary material for: Tyk2 is a tumor suppressor in colorectal cancer
Source: Oncoimmunology. 2022 Sep 26;11(1):2127271. doi: 10.1080/2162402X.2022.2127271 (PMC9519006; doi:10.1080/2162402X.2022.2127271)
Supplement: Supplemental Material [file KONI_A_2127271_SM6198.zip › Supplementary Tables.docx]

**Suppl. Table 1:** Downregulated genes in AOM/DSS induced colon tumors of Tyk2^Δ/Δ^ compared to Tyk2^+/+^ mice (downregulated > 2-fold; adjusted p-value < 0,05). log(FC): log2 fold change

| **Gene** | **log(FC)** | **adjusted p-value** |
| --- | --- | --- |
| Ms4a1 | -6,75242 | 0,00855 |
| Fcmr | -6,00875 | 0,00186 |
| Cr2 | -5,34680 | 0,00653 |
| Cd19 | -5,24907 | 0,02911 |
| Pax5 | -4,85371 | 0,02264 |
| Ffar1 | -4,83318 | 0,04836 |
| Ifi27l2a | -4,60041 | 0,00000 |
| Vip | -4,42996 | 0,00007 |
| Igfbp2 | -4,38966 | 0,00006 |
| H2-Q7, H2-Q9 | -4,30082 | 0,00000 |
| H2-Q6 | -4,10214 | 0,00000 |
| Trim30c | -4,05175 | 0,04518 |
| Ido1 | -4,02888 | 0,00000 |
| Tgtp1 | -3,89604 | 0,00000 |
| Spib | -3,87315 | 0,00304 |
| Cxcr5 | -3,81451 | 0,00549 |
| Prf1 | -3,74274 | 0,01411 |
| Gm4951 | -3,67143 | 0,00000 |
| H2-Q5 | -3,66352 | 0,00000 |
| Tnfrsf13c | -3,63151 | 0,03183 |
| Tgtp2 | -3,59113 | 0,00000 |
| Cxcl9 | -3,57699 | 0,00000 |
| Ifi44 | -3,48507 | 0,00000 |
| Fcer2a | -3,44193 | 0,00386 |
| Ly6k | -3,41891 | 0,04247 |
| Iigp1 | -3,40440 | 0,00000 |
| H2-Eb2 | -3,39200 | 0,01606 |
| Cd8a | -3,39015 | 0,00002 |
| Nlrc5 | -3,38252 | 0,00000 |
| Cd79a | -3,33550 | 0,00010 |
| Cd8b1 | -3,28842 | 0,02213 |
| Gm4841 | -3,27073 | 0,00000 |
| Ifi209 | -3,24848 | 0,00000 |
| Gvin1 | -3,24396 | 0,04485 |
| H2-Oa | -3,19010 | 0,00295 |
| Apol9a | -3,17786 | 0,00000 |
| Cd79b | -3,15582 | 0,00002 |
| Blk | -3,08824 | 0,00679 |
| Gbp2 | -3,08532 | 0,00000 |
| Olfr56 | -3,05889 | 0,00000 |
| Ifi206 | -3,05662 | 0,04171 |
| Gbp3 | -3,04875 | 0,00000 |
| Oas3 | -3,03551 | 0,00000 |
| Hspb7 | -2,95568 | 0,01835 |
| Oas2 | -2,94989 | 0,00000 |
| H2-Ob | -2,94375 | 0,00022 |
| Igtp | -2,89028 | 0,00000 |
| F830016B08Rik | -2,86878 | 0,00190 |
| Rgs13 | -2,84715 | 0,04125 |
| Btnl2 | -2,83258 | 0,01170 |
| Nkg7 | -2,81040 | 0,01170 |
| Siglecg | -2,80173 | 0,03183 |
| Ifi205 | -2,75013 | 0,00029 |
| Ifi213 | -2,74257 | 0,02641 |
| Adam33 | -2,73548 | 0,03085 |
| Des | -2,72793 | 0,00490 |
| Apol9b | -2,70669 | 0,00000 |
| Gzma | -2,68928 | 0,00015 |
| Apol10b | -2,62984 | 0,00000 |
| Gm12185 | -2,60065 | 0,00002 |
| Btnl1 | -2,58349 | 0,04841 |
| Cxcl10 | -2,58127 | 0,00000 |
| Isg15 | -2,56534 | 0,00000 |
| B2m | -2,51424 | 0,00000 |
| Slfn8 | -2,50299 | 0,00000 |
| Sox8 | -2,50224 | 0,03698 |
| Gpr18 | -2,50070 | 0,04557 |
| Rsad2 | -2,45800 | 0,00000 |
| Cnn1 | -2,44866 | 0,00098 |
| Rtp4 | -2,42859 | 0,00000 |
| Gm5431 | -2,42691 | 0,00000 |
| Oasl2 | -2,41677 | 0,00000 |
| Gcg | -2,41641 | 0,00014 |
| Ifi47 | -2,41044 | 0,00000 |
| Ifit3 | -2,40830 | 0,00000 |
| Ms4a4c | -2,39655 | 0,00043 |
| Abca13 | -2,36854 | 0,04925 |
| Tap1 | -2,36801 | 0,00000 |
| Gbp5 | -2,36114 | 0,00000 |
| H2-T10 | -2,34596 | 0,00000 |
| Ifit1 | -2,33968 | 0,00000 |
| Serpina3f | -2,31346 | 0,00000 |
| Ddx60 | -2,29029 | 0,00021 |
| Psmb8 | -2,26596 | 0,00000 |
| Ms4a4b | -2,25288 | 0,00184 |
| Gbp4 | -2,23275 | 0,00000 |
| Gm4070 | -2,23099 | 0,03042 |
| Gbp6 | -2,19594 | 0,00000 |
| Gbp10 | -2,19495 | 0,00398 |
| Ubd | -2,18822 | 0,00000 |
| Ifit3b | -2,15542 | 0,00000 |
| Far2 | -2,13445 | 0,00506 |
| H2-DMb1 | -2,11846 | 0,00000 |
| H2-T24 | -2,11437 | 0,00059 |
| Btla | -2,10382 | 0,00109 |
| Gbp2b | -2,07964 | 0,01590 |
| Pbp2 | -2,07856 | 0,04966 |
| Psmb9 | -2,05376 | 0,00000 |
| Slfn5 | -2,04606 | 0,00000 |
| Ifit2 | -2,04574 | 0,00000 |
| Gimap7 | -2,04522 | 0,00377 |
| Gbp7 | -2,02723 | 0,00000 |
| Strc | -2,00922 | 0,00000 |
| Bank1 | -1,99882 | 0,03505 |
| H2-Q4 | -1,95944 | 0,00000 |
| Trim30a | -1,95389 | 0,00000 |
| Serpine1 | -1,94834 | 0,00000 |
| Skap1 | -1,94287 | 0,02510 |
| Xaf1 | -1,93729 | 0,00000 |
| Ccl5 | -1,93570 | 0,00083 |
| Ikzf3 | -1,93367 | 0,01091 |
| Zbp1 | -1,93112 | 0,00000 |
| Klrd1 | -1,89723 | 0,00049 |
| Klrk1 | -1,89679 | 0,01006 |
| Mx1 | -1,88603 | 0,00000 |
| Phf11d | -1,87087 | 0,00000 |
| Ciita | -1,85455 | 0,00000 |
| Ptprcap | -1,85398 | 0,01170 |
| Klrb1b | -1,83900 | 0,00087 |
| Cd74 | -1,83469 | 0,00000 |
| Efcab1 | -1,83464 | 0,00855 |
| Dio2 | -1,81736 | 0,00000 |
| Art2a | -1,80917 | 0,01951 |
| H2-DMb2 | -1,79826 | 0,01592 |
| Cilp | -1,79366 | 0,01130 |
| H2-K1 | -1,79184 | 0,00000 |
| Cd274 | -1,79160 | 0,00000 |
| Irgm1 | -1,78674 | 0,00000 |
| Zfp951 | -1,77265 | 0,00109 |
| Mx2 | -1,76328 | 0,00056 |
| Gbp8 | -1,75712 | 0,00000 |
| Ctsw | -1,72760 | 0,00813 |
| Pou2af1 | -1,72666 | 0,01256 |
| Ifi203, LOC102641031 | -1,72363 | 0,00000 |
| Fosb | -1,71002 | 0,00000 |
| Insl5 | -1,70451 | 0,00014 |
| Cd22 | -1,69889 | 0,03476 |
| Sp100 | -1,69678 | 0,00000 |
| Acod1 | -1,69615 | 0,00000 |
| Sh2d2a | -1,69308 | 0,00632 |
| Gm5547 | -1,69272 | 0,01437 |
| Cd3d | -1,66578 | 0,00509 |
| Dhx58 | -1,65852 | 0,00000 |
| Fcer1a | -1,64707 | 0,04732 |
| C2 | -1,64133 | 0,00000 |
| Actg2 | -1,63614 | 0,00961 |
| Phf11b | -1,61753 | 0,01534 |
| Bst2 | -1,61742 | 0,00000 |
| Myh11 | -1,61370 | 0,01340 |
| Treml2 | -1,61111 | 0,01770 |
| Pgm5 | -1,60442 | 0,00068 |
| Trim10 | -1,60365 | 0,03953 |
| Gzmc | -1,59461 | 0,03911 |
| Serpina10 | -1,59165 | 0,00869 |
| Slfn1 | -1,58508 | 0,00004 |
| Cd3e | -1,58188 | 0,00603 |
| Herc6 | -1,57408 | 0,00000 |
| Apol6 | -1,57116 | 0,00001 |
| Rgs1 | -1,56469 | 0,00000 |
| Irf7 | -1,53504 | 0,00000 |
| Prr9 | -1,53387 | 0,00257 |
| Calhm6 | -1,51988 | 0,02390 |
| H2-T22, H2-T9 | -1,50975 | 0,00000 |
| H2-Eb1 | -1,50622 | 0,00000 |
| H2-D1 | -1,50405 | 0,00000 |
| Erdr1 | -1,50296 | 0,00136 |
| H2-Ab1 | -1,50171 | 0,00000 |
| Sema3d | -1,49951 | 0,04816 |
| Akt3 | -1,49883 | 0,00000 |
| Slc28a2 | -1,49879 | 0,03351 |
| Dsg4 | -1,49705 | 0,00000 |
| Gimap3 | -1,47495 | 0,00054 |
| Upp1 | -1,46404 | 0,00000 |
| Fcgr4 | -1,46268 | 0,00000 |
| Dpt | -1,45637 | 0,00000 |
| Mndal | -1,45410 | 0,00005 |
| Il18bp | -1,45276 | 0,00000 |
| Il5ra | -1,44903 | 0,00650 |
| Sell | -1,44844 | 0,00347 |
| Adcy5 | -1,44702 | 0,01590 |
| H2-M2 | -1,44494 | 0,00615 |
| Ppy | -1,44491 | 0,02907 |
| Lax1 | -1,43201 | 0,01415 |
| Slc15a2 | -1,42759 | 0,00297 |
| Il13ra2 | -1,41836 | 0,02102 |
| Cd3g | -1,41410 | 0,00131 |
| Casp4 | -1,41108 | 0,00000 |
| 9930111J21Rik2 | -1,40982 | 0,00511 |
| Gzmb | -1,40917 | 0,00067 |
| 2210407C18Rik | -1,39715 | 0,00000 |
| H2-DMa | -1,38808 | 0,00000 |
| Oas1b | -1,38518 | 0,00013 |
| March1 | -1,38413 | 0,00067 |
| Oas1a | -1,38267 | 0,00000 |
| Oas1g | -1,37993 | 0,00000 |
| Sp140 | -1,36239 | 0,00012 |
| Trim30d | -1,36181 | 0,00000 |
| Zfp820 | -1,36175 | 0,01423 |
| Crispld2 | -1,35769 | 0,00011 |
| Napsa | -1,35616 | 0,01292 |
| Stat1 | -1,33786 | 0,00000 |
| Slfn2 | -1,32861 | 0,00000 |
| Dcstamp | -1,32525 | 0,02750 |
| Trim5 | -1,31798 | 0,00001 |
| Klra2 | -1,31324 | 0,00083 |
| Socs1 | -1,30444 | 0,00000 |
| Synpo2 | -1,30381 | 0,00000 |
| Mreg | -1,29607 | 0,01724 |
| Tnfaip8l3 | -1,29520 | 0,04708 |
| H2-Aa | -1,29224 | 0,00000 |
| Tap2 | -1,28250 | 0,00000 |
| Erdr1 | -1,26626 | 0,00001 |
| Pyy | -1,26520 | 0,00006 |
| S100g | -1,25257 | 0,00402 |
| Usp18 | -1,24838 | 0,00000 |
| Tagln | -1,24408 | 0,00000 |
| Enpp2 | -1,23643 | 0,02150 |
| Ccl7 | -1,23439 | 0,00000 |
| Irgm2 | -1,19747 | 0,00000 |
| AI504432 | -1,19273 | 0,00712 |
| Ptgs2 | -1,19220 | 0,00000 |
| Zfp976 | -1,18793 | 0,02235 |
| Synm | -1,18742 | 0,02537 |
| Cyfip2 | -1,18561 | 0,01510 |
| Gbp9 | -1,18450 | 0,00000 |
| Zfp984 | -1,17854 | 0,00189 |
| Ube2l6 | -1,16507 | 0,00000 |
| Gpr171 | -1,16105 | 0,01498 |
| Casp12 | -1,16018 | 0,00000 |
| Il4i1, Il4i1b | -1,14784 | 0,04708 |
| Samd9l | -1,14297 | 0,00000 |
| Cd72 | -1,13758 | 0,00000 |
| Zfp960 | -1,13598 | 0,02594 |
| Pdgfd | -1,13425 | 0,00000 |
| Il7r | -1,12977 | 0,01653 |
| Ogn | -1,11674 | 0,04562 |
| St8sia6 | -1,11401 | 0,04324 |
| Cd37 | -1,10740 | 0,03012 |
| Slc10a2 | -1,09852 | 0,00000 |
| Lmod1 | -1,09814 | 0,00699 |
| Batf2 | -1,09467 | 0,00068 |
| Ptprc | -1,09396 | 0,00000 |
| Parp14 | -1,08918 | 0,00000 |
| C3 | -1,08907 | 0,00000 |
| Il2ra | -1,08870 | 0,00703 |
| Atf3 | -1,08516 | 0,00000 |
| Ifit1bl1 | -1,08261 | 0,03891 |
| AU041133 | -1,07865 | 0,02014 |
| Cd52 | -1,07579 | 0,00009 |
| Kcnb2 | -1,07440 | 0,00036 |
| Il2rb | -1,07150 | 0,00461 |
| Itgb7 | -1,06237 | 0,00327 |
| Krt90 | -1,05631 | 0,00000 |
| F13a1 | -1,05273 | 0,01510 |
| Ccr5 | -1,04890 | 0,00000 |
| Rhoh | -1,04489 | 0,01561 |
| Ly6c1 | -1,04454 | 0,00000 |
| Spn | -1,03755 | 0,00487 |
| AW112010 | -1,03363 | 0,00000 |
| Ikzf1 | -1,02847 | 0,00161 |
| Rasgrp1 | -1,01856 | 0,00821 |
| Cmpk2 | -1,01764 | 0,00002 |
| Sp110 | -1,00245 | 0,00080 |
| Lyve1 | -1,00186 | 0,00061 |
| Saa3 | -1,00111 | 0,00000 |

**Suppl. Table 2:** Upregulated genes in AOM/DSS induced colon tumors of Tyk2^Δ/Δ^ compared to Tyk2^+/+^ mice (upregulated > 2-fold; adjusted p-value < 0,05). log(FC): log2 fold change

| **Gene** | **log(FC)** | **adjusted p-value** |
| --- | --- | --- |
| Defb3 | 5,25042 | 0,01835 |
| U90926 | 4,44929 | 0,00489 |
| Pkd1l1 | 2,62481 | 0,04806 |
| Gm35279 | 2,61487 | 0,03927 |
| Vnn1 | 2,48614 | 0,00084 |
| Eya1 | 2,27761 | 0,02715 |
| Cyp1b1 | 2,10557 | 0,02924 |
| Fam181b | 2,10026 | 0,02507 |
| Hoxb3 | 2,05929 | 0,00023 |
| Hoxb5 | 2,01063 | 0,00019 |
| Obscn | 1,95428 | 0,04918 |
| Hoxb7 | 1,63552 | 0,01317 |
| Aplnr | 1,62984 | 0,00000 |
| Dnah2 | 1,61590 | 0,02936 |
| Pde11a | 1,53795 | 0,00259 |
| Ces1c | 1,28316 | 0,00042 |
| Gkn3 | 1,27349 | 0,00539 |
| Adamtsl5 | 1,25266 | 0,00379 |
| Hoxb4 | 1,24217 | 0,00070 |
| Best1 | 1,12331 | 0,00684 |
| Abcc6 | 1,10004 | 0,00097 |
| Rec8 | 1,09482 | 0,00000 |
| Agmat | 1,05465 | 0,01560 |

**Suppl. Table 3:** Downregulated genes in AOM/DSS induced colon tumors of Tyk2^ΔIEC^ compared to Tyk2^fl/fl^ mice (downregulated > 2-fold; adjusted p-value < 0,05). log(FC): log2 fold change

| **Gene** | **log(FC)** | **adjusted p‑value** |
| --- | --- | --- |
| ATP6 | -24,22184 | 0,00000 |
| Xist | -10,91511 | 0,00000 |
| Gm7266 | -5,13788 | 0,00014 |
| Gm6192 | -5,12511 | 0,00000 |
| Gm6440 | -4,93052 | 0,00000 |
| Serpina3k | -4,52743 | 0,01038 |
| Rpl7a-ps10 | -4,42542 | 0,00106 |
| Gm7429 | -4,22142 | 0,00207 |
| Rps12-ps19 | -3,96893 | 0,00255 |
| Gm13181 | -3,95118 | 0,00555 |
| Igkv4-74 | -3,92336 | 0,01002 |
| Gm14113 | -3,90803 | 0,00317 |
| Gm8268 | -3,88028 | 0,00593 |
| Gm7436 | -3,85678 | 0,00763 |
| Rps8-ps1 | -3,82691 | 0,00366 |
| Gm8919 | -3,81845 | 0,00536 |
| Gm6190 | -3,74121 | 0,00823 |
| Prr30 | -3,39039 | 0,02087 |
| Igkv14-100 | -3,33978 | 0,00766 |
| Gm15191 | -3,28096 | 0,00391 |
| Rps3a2 | -3,28019 | 0,00011 |
| Gm12722 | -3,13612 | 0,03150 |
| Gm9061 | -3,06259 | 0,03286 |
| Rpl13-ps6 | -2,96652 | 0,04064 |
| Gm9497 | -2,96064 | 0,00013 |
| H19 | -2,89791 | 0,00420 |
| Gm6931 | -2,84135 | 0,00437 |
| Gm8692 | -2,77972 | 0,00000 |
| Scgb3a1 | -2,76118 | 0,04982 |
| Gm18913 | -2,74326 | 0,01203 |
| Rpl23a-ps3 | -2,67441 | 0,02713 |
| Gm6851 | -2,66399 | 0,01011 |
| Rps2-ps8 | -2,66082 | 0,00062 |
| Gm8925 | -2,65495 | 0,01663 |
| Gm5511 | -2,61187 | 0,03264 |
| Gm5944 | -2,55784 | 0,00781 |
| Rps7-ps3 | -2,54413 | 0,00000 |
| Aqp5 | -2,53263 | 0,00003 |
| Gm6771 | -2,51729 | 0,00356 |
| Gm5047 | -2,50797 | 0,00050 |
| Rps6-ps4 | -2,48363 | 0,00000 |
| Gm5297 | -2,45852 | 0,00914 |
| Gm7384 | -2,45219 | 0,03002 |
| Llcfc1 | -2,42386 | 0,00541 |
| Gdf6 | -2,37529 | 0,00064 |
| Gm6368 | -2,37475 | 0,00000 |
| Gm4799 | -2,36659 | 0,01369 |
| Gm13498 | -2,34591 | 0,01189 |
| Gm4823 | -2,31922 | 0,03742 |
| Ighv14-3 | -2,31150 | 0,01818 |
| Defa40 | -2,30530 | 0,04804 |
| Orm3 | -2,29767 | 0,01149 |
| Gm11453 | -2,20957 | 0,02628 |
| Gm10566 | -2,20660 | 0,01221 |
| Gm5424 | -2,17408 | 0,00000 |
| Gm6023 | -2,11973 | 0,00654 |
| Rpl32l | -2,11205 | 0,03843 |
| Ndufs6b | -2,09436 | 0,00694 |
| Gm8825 | -2,07480 | 0,01832 |
| Gm7507 | -2,06325 | 0,00475 |
| Gm6863 | -2,05328 | 0,00000 |
| Gm4929 | -2,04269 | 0,00609 |
| Gm6204 | -1,97193 | 0,00000 |
| Tubb4b-ps1 | -1,96748 | 0,02014 |
| Gm9493 | -1,91874 | 0,00248 |
| Gm4948 | -1,83481 | 0,04436 |
| Gm2467 | -1,81219 | 0,02217 |
| Gm10288 | -1,79944 | 0,00470 |
| Gm12669 | -1,79719 | 0,00001 |
| Spp1 | -1,79298 | 0,00000 |
| Bc1 | -1,78996 | 0,00024 |
| Gm5430 | -1,77136 | 0,04102 |
| Gm5835 | -1,75168 | 0,00000 |
| Madcam1 | -1,74598 | 0,00064 |
| Gm8623 | -1,74422 | 0,02581 |
| Prss35 | -1,73192 | 0,00005 |
| Gm7899 | -1,71782 | 0,03827 |
| Map2 | -1,71569 | 0,00894 |
| Gm5905 | -1,70656 | 0,00000 |
| Col9a3 | -1,69191 | 0,00017 |
| Gm23645 | -1,67542 | 0,04263 |
| Gm6278 | -1,67356 | 0,00808 |
| Gm5837 | -1,66716 | 0,04862 |
| Btf3-ps4 | -1,66233 | 0,02965 |
| Gm15501 | -1,65824 | 0,00000 |
| Gm5913 | -1,64861 | 0,00000 |
| Gm6394 | -1,64580 | 0,00000 |
| Gm11221 | -1,62379 | 0,02446 |
| Gm10224 | -1,62320 | 0,02789 |
| Depp1 | -1,61657 | 0,00000 |
| Angpt4 | -1,60904 | 0,00000 |
| Gm4366 | -1,60672 | 0,00000 |
| Gm5921 | -1,60388 | 0,04246 |
| Kif19a | -1,60080 | 0,00391 |
| Gm12020 | -1,59957 | 0,03217 |
| Gm12366 | -1,59461 | 0,01779 |
| Gm8130 | -1,58771 | 0,00066 |
| Gm10177 | -1,57184 | 0,00069 |
| Gm29257 | -1,56124 | 0,00129 |
| Gm2614 | -1,55639 | 0,00672 |
| Gm4540 | -1,54685 | 0,00097 |
| Rps3a3 | -1,54379 | 0,00037 |
| Tpsb2 | -1,54296 | 0,00453 |
| Rpl18-ps2 | -1,53717 | 0,00001 |
| Cntfr | -1,52911 | 0,01242 |
| Rps23-ps1 | -1,52873 | 0,00185 |
| Defa3 | -1,50737 | 0,00724 |
| Eif3j2 | -1,49232 | 0,00008 |
| Klhl32 | -1,48490 | 0,00398 |
| Rps12-ps10 | -1,48483 | 0,00205 |
| Col9a1 | -1,47899 | 0,00000 |
| Snhg9 | -1,47649 | 0,00159 |
| Serpinf2 | -1,46536 | 0,00024 |
| Fam71f2 | -1,46218 | 0,02700 |
| Gm5586 | -1,44632 | 0,00000 |
| Gm2735 | -1,44534 | 0,00470 |
| Gm10131 | -1,43391 | 0,02316 |
| Medag | -1,42867 | 0,00000 |
| Rhox5 | -1,42261 | 0,00765 |
| Rpsa-ps10 | -1,42138 | 0,00002 |
| Defa21 | -1,42096 | 0,00095 |
| Twist1 | -1,41460 | 0,00370 |
| Gm13680 | -1,40575 | 0,02156 |
| Comp | -1,40263 | 0,00462 |
| Draxin | -1,39806 | 0,02017 |
| Wfdc18 | -1,39478 | 0,00000 |
| Wnt2 | -1,38621 | 0,03736 |
| Defa34 | -1,38143 | 0,01746 |
| Gm8276 | -1,37903 | 0,00064 |
| Rpsa-ps4 | -1,37250 | 0,03311 |
| Acat3 | -1,35727 | 0,00370 |
| Gm11675 | -1,35579 | 0,00664 |
| Sirpb1b | -1,33693 | 0,04065 |
| Gm17893 | -1,33318 | 0,01942 |
| Gm5778 | -1,32566 | 0,00139 |
| Rpl19-ps11 | -1,32060 | 0,03509 |
| Defa17 | -1,31950 | 0,00000 |
| Rps18-ps3 | -1,31812 | 0,02838 |
| Capn6 | -1,31258 | 0,02700 |
| Gm4691 | -1,31154 | 0,00787 |
| Rspo4 | -1,30511 | 0,02304 |
| Rps15a-ps7 | -1,30087 | 0,00228 |
| Gm20900 | -1,30007 | 0,04677 |
| Defa30 | -1,29997 | 0,00713 |
| Ak5 | -1,29487 | 0,01079 |
| Rps27rt | -1,28966 | 0,00208 |
| Bloc1s1 | -1,28066 | 0,00251 |
| Gm13689 | -1,27551 | 0,03283 |
| Afp | -1,27023 | 0,02867 |
| Defa22 | -1,26910 | 0,00156 |
| Serpind1 | -1,26420 | 0,01254 |
| Gm6254 | -1,25609 | 0,03284 |
| Mfap4 | -1,23378 | 0,00085 |
| Rpl9-ps6 | -1,23214 | 0,00000 |
| Gpi-ps | -1,21193 | 0,00006 |
| Rpl10-ps3 | -1,21012 | 0,00005 |
| Hrct1 | -1,20812 | 0,04131 |
| Gm3571 | -1,20652 | 0,00234 |
| Trabd2b | -1,19767 | 0,00004 |
| Gm16580 | -1,19709 | 0,00000 |
| Lum | -1,18919 | 0,00001 |
| Ccl7 | -1,17743 | 0,00030 |
| Cibar2 | -1,16667 | 0,00843 |
| Gm16061 | -1,16344 | 0,01705 |
| Rgs1 | -1,15491 | 0,00904 |
| Fzd9 | -1,15386 | 0,00018 |
| Megf11 | -1,15030 | 0,00051 |
| Gm14586 | -1,14643 | 0,00004 |
| Ppp1ccb | -1,14544 | 0,00006 |
| Ch25h | -1,13803 | 0,00091 |
| Rflna | -1,12591 | 0,04029 |
| Bex4 | -1,12220 | 0,00311 |
| Rims2 | -1,11260 | 0,00117 |
| Sfrp1 | -1,10636 | 0,00049 |
| Npb | -1,09498 | 0,00001 |
| Gm8730 | -1,09484 | 0,00714 |
| Fgf3 | -1,09480 | 0,00042 |
| Gm5637 | -1,08465 | 0,00027 |
| Atp1a2 | -1,08055 | 0,00001 |
| Gm4332 | -1,06992 | 0,00000 |
| Gm6136 | -1,06935 | 0,00000 |
| Rgcc | -1,06808 | 0,00000 |
| Cdh4 | -1,06573 | 0,03579 |
| Gm7536 | -1,06398 | 0,00802 |
| Spns3 | -1,06307 | 0,02229 |
| Bcl2a1b | -1,06088 | 0,00008 |
| Pde11a | -1,05558 | 0,02859 |
| Ccna1 | -1,05364 | 0,02088 |
| Lrg1 | -1,05139 | 0,02203 |
| Gm9794 | -1,04841 | 0,00000 |
| Rpl10a-ps1 | -1,04817 | 0,00000 |
| Gja5 | -1,04713 | 0,00502 |
| Rps10-ps1 | -1,03565 | 0,00060 |
| Hmgcs2 | -1,03441 | 0,00002 |
| Nrtn | -1,02833 | 0,00000 |
| Gm9625 | -1,02273 | 0,00000 |
| Gm11249 | -1,02152 | 0,01386 |
| Rpl37rt | -1,01979 | 0,00000 |
| C030013C21Rik | -1,01830 | 0,02031 |
| Mfap2 | -1,01508 | 0,00024 |
| Tmem252 | -1,01355 | 0,00123 |
| Crlf1 | -1,01054 | 0,00302 |
| Gm10250 | -1,00911 | 0,01609 |
| Ccl22 | -1,00772 | 0,00787 |
| Tlx2 | -1,00715 | 0,04311 |
| Nkx2-3 | -1,00567 | 0,00035 |
| Pdcd1 | -1,00525 | 0,00721 |
| Tox2 | -1,00488 | 0,02870 |
| Gm5805 | -1,00039 | 0,00004 |

**Suppl. Table 4:** Upregulated genes in AOM/DSS induced colon tumors of Tyk2^ΔIEC^ compared to Tyk2^fl/fl^ mice (upregulated > 2-fold; adjusted p-value < 0,05). log(FC): log2 fold change

| **Gene** | **log(FC)** | **adjusted p‑value** |
| --- | --- | --- |
| Slc9b1 | 6,53336 | 0,00008 |
| Gm20426 | 6,35250 | 0,00000 |
| Vmn1r53 | 6,21623 | 0,00000 |
| Myo18b | 4,97626 | 0,00000 |
| Gm15446 | 4,68097 | 0,00002 |
| Psca | 4,62502 | 0,00073 |
| Spink5 | 4,44659 | 0,00108 |
| Cyp2f2 | 4,29208 | 0,00081 |
| Ltf | 3,92600 | 0,00004 |
| Capn11 | 3,75119 | 0,00215 |
| Calcb | 2,55730 | 0,00005 |
| Ppbp | 2,51332 | 0,00630 |
| Padi1 | 2,46380 | 0,02278 |
| Kcnab1 | 2,42823 | 0,00513 |
| Apela | 2,39122 | 0,00302 |
| Olfm4 | 2,34786 | 0,00129 |
| Igkv8-27 | 2,29584 | 0,03655 |
| S100g | 2,26132 | 0,00839 |
| Neb | 2,23166 | 0,00212 |
| Sv2b | 2,22341 | 0,00262 |
| Uts2r | 2,20233 | 0,02022 |
| Ibsp | 2,18475 | 0,00069 |
| Olfr111 | 2,17720 | 0,01207 |
| Ido1 | 2,14788 | 0,00495 |
| Gm7859 | 2,13407 | 0,00212 |
| Lypd2 | 2,12062 | 0,03195 |
| Krt4 | 2,11759 | 0,00000 |
| Tgtp1 | 2,08855 | 0,00007 |
| Hes5 | 2,08783 | 0,02070 |
| Igf2bp1 | 2,08142 | 0,00245 |
| Sstr1 | 2,06922 | 0,00005 |
| 4933433G15Rik | 2,04745 | 0,00015 |
| Cps1 | 2,04452 | 0,01961 |
| Pyy | 2,04286 | 0,00399 |
| Mir6942 | 2,03346 | 0,00747 |
| Gbp10 | 2,01950 | 0,00714 |
| A730082K24Rik | 2,01798 | 0,01389 |
| Adh7 | 1,98133 | 0,00708 |
| Tex19.1 | 1,97280 | 0,00300 |
| Dsg4 | 1,96209 | 0,00007 |
| Ugt1a1 | 1,95867 | 0,04459 |
| Gm17783 | 1,95398 | 0,03942 |
| 2010106E10Rik | 1,94223 | 0,04039 |
| H2-Q5 | 1,93334 | 0,00030 |
| Krt15 | 1,88566 | 0,00000 |
| Sez6l | 1,83790 | 0,00212 |
| Sytl5 | 1,82545 | 0,00620 |
| Ntm | 1,82164 | 0,04709 |
| Ap3b2 | 1,79163 | 0,00138 |
| Strc | 1,76456 | 0,02894 |
| Klhdc9 | 1,76444 | 0,01405 |
| Sprr2a2 | 1,75632 | 0,01545 |
| H2-Q7, H2-Q9 | 1,74817 | 0,00538 |
| Pkd1l1 | 1,74780 | 0,01831 |
| Ppef1 | 1,72781 | 0,00413 |
| Trim30c | 1,71121 | 0,00993 |
| Pcdhgb1 | 1,70085 | 0,01614 |
| H2-Q6 | 1,69309 | 0,01339 |
| Tgtp2 | 1,68872 | 0,00064 |
| Cnr1 | 1,68698 | 0,00010 |
| Ifit2 | 1,67776 | 0,00000 |
| Clca4a | 1,66348 | 0,02859 |
| Efhb | 1,65679 | 0,01191 |
| Cyp2b10 | 1,65533 | 0,00000 |
| Mmp3 | 1,64761 | 0,00055 |
| Ces2a | 1,63778 | 0,00561 |
| Fmo2 | 1,62398 | 0,00000 |
| Btnl6 | 1,61731 | 0,00005 |
| Gbp2 | 1,59433 | 0,00316 |
| Avpr1b | 1,56129 | 0,03863 |
| Tnn | 1,55634 | 0,03403 |
| Elf5 | 1,55557 | 0,04354 |
| Ddx60 | 1,55396 | 0,00000 |
| Upk3bl | 1,55171 | 0,03079 |
| Lncpint | 1,54102 | 0,00212 |
| Shh | 1,51437 | 0,01098 |
| Tnfrsf14 | 1,50240 | 0,00000 |
| Tubal3 | 1,49794 | 0,02528 |
| Kcnh7 | 1,48644 | 0,00365 |
| Pcdhga5 | 1,43033 | 0,01705 |
| Nlrc5 | 1,41109 | 0,00620 |
| Mettl7a2 | 1,40539 | 0,04334 |
| Apol10b | 1,39624 | 0,00029 |
| Rasgrf2 | 1,36831 | 0,02829 |
| Lypd8l | 1,36615 | 0,00000 |
| 4930426D05Rik | 1,35969 | 0,00000 |
| Cyp2c68 | 1,35342 | 0,01441 |
| Enpp3 | 1,35013 | 0,00138 |
| Gm16063 | 1,34956 | 0,03133 |
| Adh6a | 1,34941 | 0,00238 |
| Klhl11 | 1,34684 | 0,00079 |
| Oas3 | 1,34599 | 0,00001 |
| 9530026P05Rik | 1,34374 | 0,00793 |
| Lancl3 | 1,34322 | 0,00057 |
| Per2 | 1,33887 | 0,00000 |
| Nos1ap | 1,33807 | 0,00000 |
| Nr1h5 | 1,33760 | 0,00653 |
| Lrrn3 | 1,33601 | 0,02915 |
| Gm4951 | 1,33224 | 0,00641 |
| Klhl14 | 1,33004 | 0,00000 |
| Gm12185 | 1,32547 | 0,00318 |
| Tppp3 | 1,31728 | 0,00000 |
| Apol9a | 1,30896 | 0,00011 |
| F5 | 1,30730 | 0,00008 |
| H60c | 1,30604 | 0,00006 |
| Lrrc31 | 1,30129 | 0,00000 |
| Apol6 | 1,29876 | 0,00150 |
| Rorc | 1,28915 | 0,00000 |
| Nrg4 | 1,28511 | 0,01137 |
| Dach2 | 1,28353 | 0,00000 |
| Efhd1 | 1,28031 | 0,03680 |
| Ccdc170 | 1,27172 | 0,00175 |
| Sectm1b | 1,26918 | 0,00252 |
| Lcor | 1,26842 | 0,00000 |
| Gm22009 | 1,26664 | 0,02183 |
| Grb14 | 1,26623 | 0,00259 |
| 9930111J21Rik1 | 1,26290 | 0,02688 |
| Iigp1 | 1,26189 | 0,02191 |
| Pcdhgb2 | 1,25928 | 0,00060 |
| A1cf | 1,25486 | 0,00001 |
| Srgap3 | 1,24974 | 0,00000 |
| Gm609 | 1,23834 | 0,00555 |
| Htra4 | 1,23652 | 0,01510 |
| Gm26671 | 1,23184 | 0,01065 |
| Gprc5c | 1,22566 | 0,00091 |
| Gdpd2 | 1,22493 | 0,02832 |
| Gm16011 | 1,22222 | 0,00558 |
| Ciita | 1,22166 | 0,00069 |
| Exph5 | 1,21484 | 0,00000 |
| Gbp3 | 1,21165 | 0,00004 |
| Ptprn2 | 1,20937 | 0,00840 |
| Tlr9 | 1,20763 | 0,02141 |
| Trim66 | 1,20740 | 0,04217 |
| Igtp | 1,19938 | 0,00064 |
| Fmo1 | 1,19928 | 0,00069 |
| Pcdhb6 | 1,19635 | 0,03396 |
| Ccdc33 | 1,18364 | 0,00162 |
| Gm16001 | 1,17815 | 0,00123 |
| Per3 | 1,17531 | 0,00000 |
| Abcg8 | 1,17221 | 0,00011 |
| Ifit3 | 1,16287 | 0,00030 |
| Gm24616 | 1,15078 | 0,00290 |
| Tmem132c | 1,14720 | 0,00985 |
| Cacna2d2 | 1,14177 | 0,01477 |
| Cxcl10 | 1,13980 | 0,00651 |
| Greb1l | 1,13934 | 0,03424 |
| Pcdh19 | 1,13778 | 0,00022 |
| Plag1 | 1,13735 | 0,04101 |
| Eci3 | 1,13647 | 0,00358 |
| Adnp | 1,12808 | 0,00083 |
| Syne3 | 1,12484 | 0,00017 |
| Gm24494 | 1,12367 | 0,00311 |
| Slc6a12 | 1,12325 | 0,00064 |
| Mid1 | 1,12235 | 0,00000 |
| Pdpr | 1,12089 | 0,00001 |
| Dennd4c | 1,11325 | 0,00000 |
| Tef | 1,11234 | 0,00000 |
| Olfr56 | 1,10958 | 0,01123 |
| H2-T10 | 1,10616 | 0,00196 |
| Apol9b | 1,10452 | 0,00287 |
| 6030443J06Rik | 1,09982 | 0,00000 |
| Sprr2b | 1,09833 | 0,02202 |
| Cd274 | 1,08019 | 0,00000 |
| Pitpnm3 | 1,07897 | 0,00000 |
| Ifit1 | 1,07799 | 0,00035 |
| Tap1 | 1,07316 | 0,00029 |
| Hhip | 1,07256 | 0,02365 |
| Atp7a | 1,06855 | 0,00000 |
| Soga1 | 1,06774 | 0,00002 |
| Vegfd | 1,06564 | 0,00700 |
| Atm | 1,06549 | 0,00000 |
| Gbp6 | 1,06403 | 0,00498 |
| Kcnu1 | 1,05939 | 0,00005 |
| Gm5431 | 1,05721 | 0,00455 |
| Mark1 | 1,05014 | 0,00000 |
| Uprt | 1,04598 | 0,00001 |
| Foxn1 | 1,04427 | 0,00094 |
| Vav3 | 1,03839 | 0,00008 |
| Gm22571 | 1,03719 | 0,01614 |
| Ifi44 | 1,03693 | 0,00005 |
| Slc10a5 | 1,03006 | 0,00046 |
| 9030619P08Rik | 1,02920 | 0,03003 |
| Cpm | 1,02785 | 0,00000 |
| Rassf9 | 1,01673 | 0,00008 |
| Cyyr1 | 1,01491 | 0,00729 |
| Pcnx2 | 1,00927 | 0,04138 |
| Efcab8 | 1,00595 | 0,03460 |
| Papln | 1,00472 | 0,00079 |
